# A positive cytokine/chemokine feedback loop establishes plasmacytoid DC–driven autoimmune pancreatitis in IgG4-related disease

**DOI:** 10.1172/jci.insight.167910

**Published:** 2024-09-12

**Authors:** Akane Hara, Tomohiro Watanabe, Kosuke Minaga, Tomoe Yoshikawa, Masayuki Kurimoto, Ikue Sekai, Yasuhiro Masuta, Ryutaro Takada, Yasuo Otsuka, Ken Kamata, Shiki Takamura, Masatoshi Kudo, Warren Strober

**Affiliations:** 1 Department of Gastroenterology and Hepatology, Kindai University Faculty of Medicine, Osaka-Sayama, Osaka, Japan.; 2 Laboratory for Immunological Memory, RIKEN IMS Center for Integrative Medical Science, Yokohama, Japan.; 3 Mucosal Immunity Section, Laboratory of Host Defenses, National Institute of Allergy and Infectious Diseases, NIH, Bethesda, Maryland, USA.

**Keywords:** Autoimmunity, Gastroenterology, Chemokines, Cytokines

## Abstract

The pathogenesis of the murine model of autoimmune pancreatitis associated with IgG4-related disease (AIP/IgG4-RD) induced by administration of polyinosinic-polycytidylic acid (poly[I:C]) is incompletely understood. While it is known that murine and human AIP/IgG4-RD is driven by plasmacytoid dendritic cells (pDCs) producing IFN-α, the origin of these cells and their relation to effector T cells is not known. Here, we show that murine AIP was initiated by TLR3-bearing conventional DCs in the uninflamed pancreas whose activation by the TLR3 ligand poly(I:C) caused IFN-α, CXCL9, and CXCL10 secretion. This, in turn, induced pancreatic recruitment of CXCR3^+^ T cells and these T cells, via their secretion of CCL25, facilitated migration of pDCs bearing CCR9 into the pancreas. This established a feedback loop anchored by the now dominant pDC production of IFN-α and the continued CXCR3^+^ T cell facilitation of pDC migration. Remarkably, the interaction between CXCR3^+^ T cells and pDCs also existed at the functional level since this interaction enhanced the production of CCL25 and IFN-α by CXCR3^+^ T cells and pDCs, respectively. Evidence presented here that a similar disease mechanism was present in human AIP/IgG4-RD creates new avenues of disease treatment.

## Abstract 2

A positive feedback loop consisting of IFN-alpha CXCL9, CXCL10, and CCL25 mediates experimental and human autoimmune pancreatitis.

## Graphical abstract

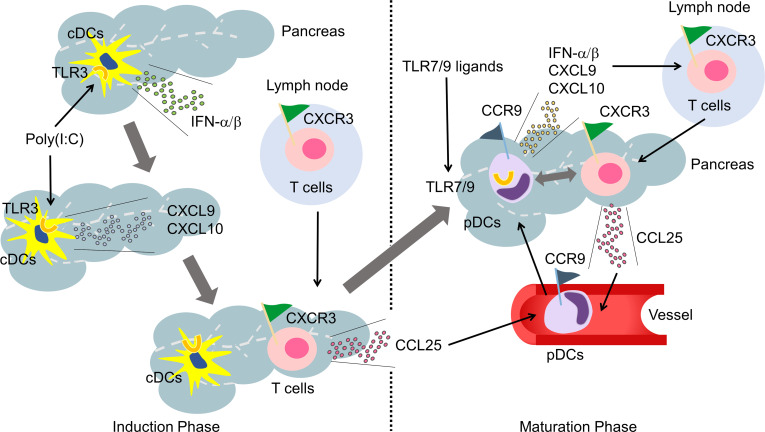

## Introduction

Clinicopathological analysis of autoimmune pancreatitis (AIP) and other organ inflammations accompanying AIP such as autoimmune sialadenitis and autoimmune cholangitis indicated that these autoimmune disorders are organ-specific manifestations of a recently established systemic autoimmune disorder now called IgG4-related disease (IgG4-RD) (1–3). In recent studies, the pathogenesis of this form of autoimmunity has been explored in a murine model of AIP phenotypically similar to that in the human AIP associated with IgG4-RD that is induced in MRL/MpJ mice by repeated administration of polyinosinic-polycytidylic acid (poly[I:C]). A key finding was that inflamed pancreatic tissue in this model was replete with plasmacytoid dendritic cells (pDCs) producing IFN-α and IL-33 and that depletion of these cells or blockade of IFN-α– or IL-33–mediated signaling pathways almost completely protected the mice from the development of pancreatitis and its fibrotic sequelae (3–6). These studies thus provided strong evidence that experimental AIP is dependent on activated pDCs producing IFN-α and IL-33. Inasmuch as such pDCs are localized in the pancreas of patients with AIP/IgG4-RD and the patients exhibit elevated serum concentrations of IFN-α and IL-33, this conclusion also applies to the pathogenesis of human AIP/IgG4-RD (3–5, 7).

Important, but as yet unanswered, questions concerning the murine model described above is the origin of the major pathogenic cells, pDCs, and how these cells interact with effector T cells causing disease. The first part of this question relating to pDCs arises from the fact that pancreatic inflammation is initiated by a Toll-like receptor 3 (TLR3) ligand (poly[I:C]), yet the disease-sustaining pDCs express only low levels of TLR3 (8); thus, the cell initiating murine AIP is not yet defined. The second part of this question concerning the T cells relates to previous evidence that AIP/IgG4-RD is accompanied by the generation of a multifaceted T cell array and it is not clear how the various T cells comprising this array contribute to the disease process (9–16). Given the importance of the pDC-driven type I IFN response in both murine and human AIP/IgG4-RD, one might predict that T helper type 1 (Th1) and/or follicular helper T type 1 (Tfh1) cells are a dominant part of the T cell response and indeed enumeration of the various types of T cells found in lesional tissue associated with AIP/IgG4-RD shows that IFN-γ–producing T cells comprise a large fraction of the total T cell population present in such tissue (9, 15–17). It therefore becomes important to define the nature of the T cells initially generated in the murine model and their relation to the overall disease process.

In the present study, we addressed these questions and found that pancreatic TLR3^+^ conventional DCs (cDCs) are the poly(I:C)-responsive cells that initiate murine AIP. Moreover, we found that these cells, via their secretion of C-X-C motif chemokine ligand 9 and 10 (CXCL9 and CXCL10), attract C-X-C motif chemokine receptor 3^+^ (CXCR3^+^) T cells into the pancreas. Under the influence of IFN-α secreted at this site, these T cells produce C-C motif chemokine ligand 25 (CCL25), a chemokine ligand that attracts disease-sustaining pDCs expressing C-C chemokine receptor 9 (CCR9) into the pancreas. This train of events sets up a positive chemokine and cytokine feedback loop wherein pancreatic pDCs, upon stimulation with TLR9 ligands, become the source of the IFN-α that induces further rounds of migration of CXCR3^+^ T cells and pDCs into the pancreas. We also found that CXCR3^+^ T cell interaction with pDCs is critical for high-level IFN-α production by pancreatic pDCs; thus, these T cells have the dual roles of both attracting pDCs into the pancreas and stimulating pDCs that have arrived at this site. The cytokine and chemokine components of the AIP-generating feedback loop found in the murine AIP model are elevated in the serum of patients with AIP/IgG4-RD; thus, it is possible that a similar feedback loop is operative in human AIP/IgG4-RD.

## Results

### The uninflamed pancreas harbors cDCs that express high levels of TLR3.

As alluded to in the Introduction, the model of AIP/IgG4-RD in MRL/MpJ mice induced by repeated (16-fold) administration of poly(I:C) is mainly driven by activated pDCs (3–5). This introduces a conundrum because in previous studies, while pDCs isolated from uninflamed splenic tissue express high levels of TLR7 and TLR9, they lack expression of the TLR capable of responding to poly(I:C), TLR3; on the other hand, in the same studies, isolated CD11c^+^ cDCs were found to express high levels of TLR3 (8). To determine whether a similar situation exists in uninflamed pancreatic tissue of MRL/MpJ mice, pDCs and cDCs in pancreatic mononuclear cells (PMNCs) from these mice were purified by flow cytometric sorting on the basis of PDCA-1^hi^B220^lo^ and CD11c^hi^MHC class II^hi^ surface markers, respectively, and then analyzed by quantitative reverse transcription PCR (qPCR) to determine mRNA expression of *Tlr3* and *Tlr9* (Supplemental Figure 1A; supplemental material available online with this article; https://doi.org/10.1172/jci.insight.167910DS1). We found that whereas both pancreatic cDCs and pDCs express higher levels of *Tlr3* mRNA than counterpart cells in the spleen, cDCs are by far the main TLR3-expressing cells in uninflamed pancreatic tissue (Supplemental Figure 1B). In addition, both splenic and pancreatic pDCs from uninflamed tissues expressed only modestly higher levels of *Tlr9* mRNA than splenic cDCs (Supplemental Figure 1B). These results indicate that cDCs are more likely than pDCs to be the major cell initiating murine AIP upon repeated administration of poly(I:C).

### Blockade of TLR3 inhibits the development of experimental AIP.

We next determined whether the development of experimental AIP requires recognition of poly(I:C) by TLR3-expressing cells in the uninflamed pancreas. To this end, we performed TLR3 inhibition studies utilizing a well-studied competitive inhibitor of double-stranded RNA (dsRNA) binding to TLR3 [(*R*)-2-(3-chloro-6-fluorobenzo(b)thiophene-2-carboxamido)-3-phenylpropanoic acid] to determine whether inhibition of TLR3 signaling blocks development of AIP; importantly, this inhibitor has previously been shown not to inhibit signaling by other TLRs, including signaling by TLR7 (18, 19).

**Figure 1:**
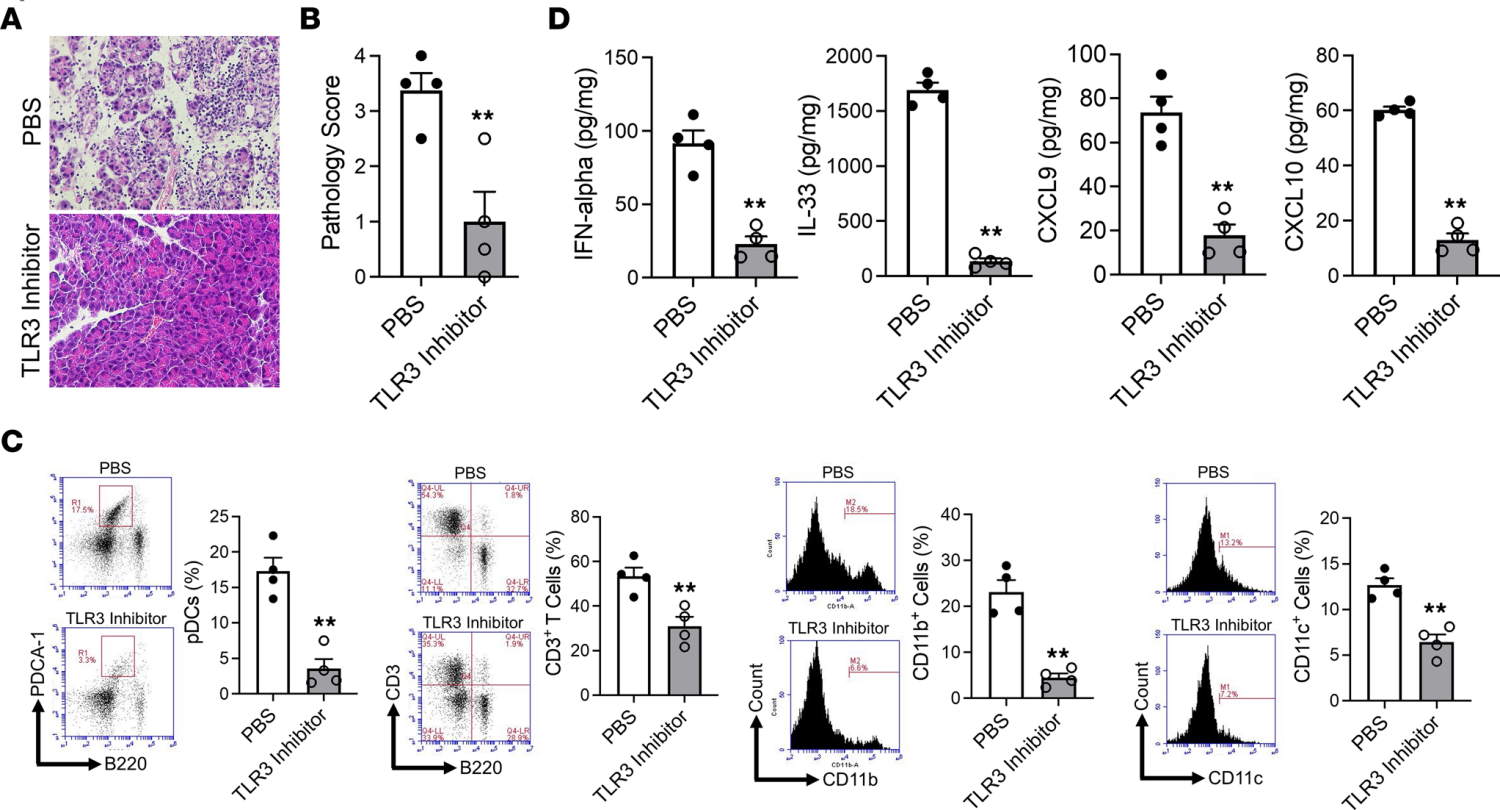
Blockade of TLR3 by inhibitors prevents the development of experimental autoimmune pancreatitis. MRL/MpJ mice were administered poly(I:C) by intraperitoneal injection twice a week for a total of 16 times to induce experimental autoimmune pancreatitis (AIP). Each poly(I:C) injection was preceded by intraperitoneal injection of saline (PBS, *n* = 4) or TLR3/dsRNA binding inhibitor (1 mg, *n* = 4). After sacrifice at 3 hours following the final set of injections, pancreases were removed and analyzed as indicated. (**A** and **B**) Hematoxylin and eosin staining of the pancreatic tissues and pathological scores of induced AIP in the 2 groups. Original magnification, ×400. (**C**) Percentages of plasmacytoid DCs (pDCs), CD3^+^ T cells, CD11b^+^ myeloid cells, and CD11c^+^ DCs within pancreatic mononuclear cells, as determined by flow cytometric analyses. pDCs were defined as PDCA-1^+^B220^lo^ cells. Left panels: Representative cytometric analysis. Right panels: Bar graphs of cumulative results. (**D**) Concentrations of IFN-α, IL-33, CXCL9, and CXCL10 in protein extracts of pancreatic tissues from mice without and with TLR3 inhibitor administration, as determined by ELISA. Each dot corresponds to the value in 1 mouse. Statistical analyses were performed using an unpaired, 2-tailed Student’s *t* test. Results are expressed as mean ± SEM. **P < 0.01.

We found that whereas repeated administration of both poly(I:C) and saline control to MRL/MpJ mice led, as expected, to the development of AIP characterized histologically by destruction of pancreatic acinar architecture, infiltration of immune cells, and massive fibrosis, repeated injections of both poly(I:C) and the inhibitor of TLR3 signaling led to only minor histologic changes of this kind (Figure 1, A and B). By flow cytometric analysis of PMNCs from mice subjected to TLR3 blockade and control mice (Supplemental Figure 2A), we also found that blockade of TLR3 signaling led to a great reduction in the cellular immune response previously shown to cause AIP, i.e., a reduction in the pancreatic accumulation of pDCs (defined as PDCA-1^+^B220^lo^ cells) and CD3^+^ T cells (Figure 1C). In addition, it led to reduction in the accumulation of CD11b^+^ myeloid cells and CD11c^+^ DCs (Figure 1C) and to a reduction in their CD11b and CD11c mean fluorescence intensities (MFIs) (Supplemental Figure 2B). Likewise, blockade of TLR3 signaling was accompanied by decreased pancreatic expression of IFN-α and IL-33, both of which have been shown to be produced by infiltrating pDCs in this model of AIP (3–6) (Figure 1D). Finally, blockade led to decreased pancreatic expression of CXCL9 and CXCL10, Th1 chemokines dependent on type I IFN responses (Figure 1D) (20).

**Figure 2:**
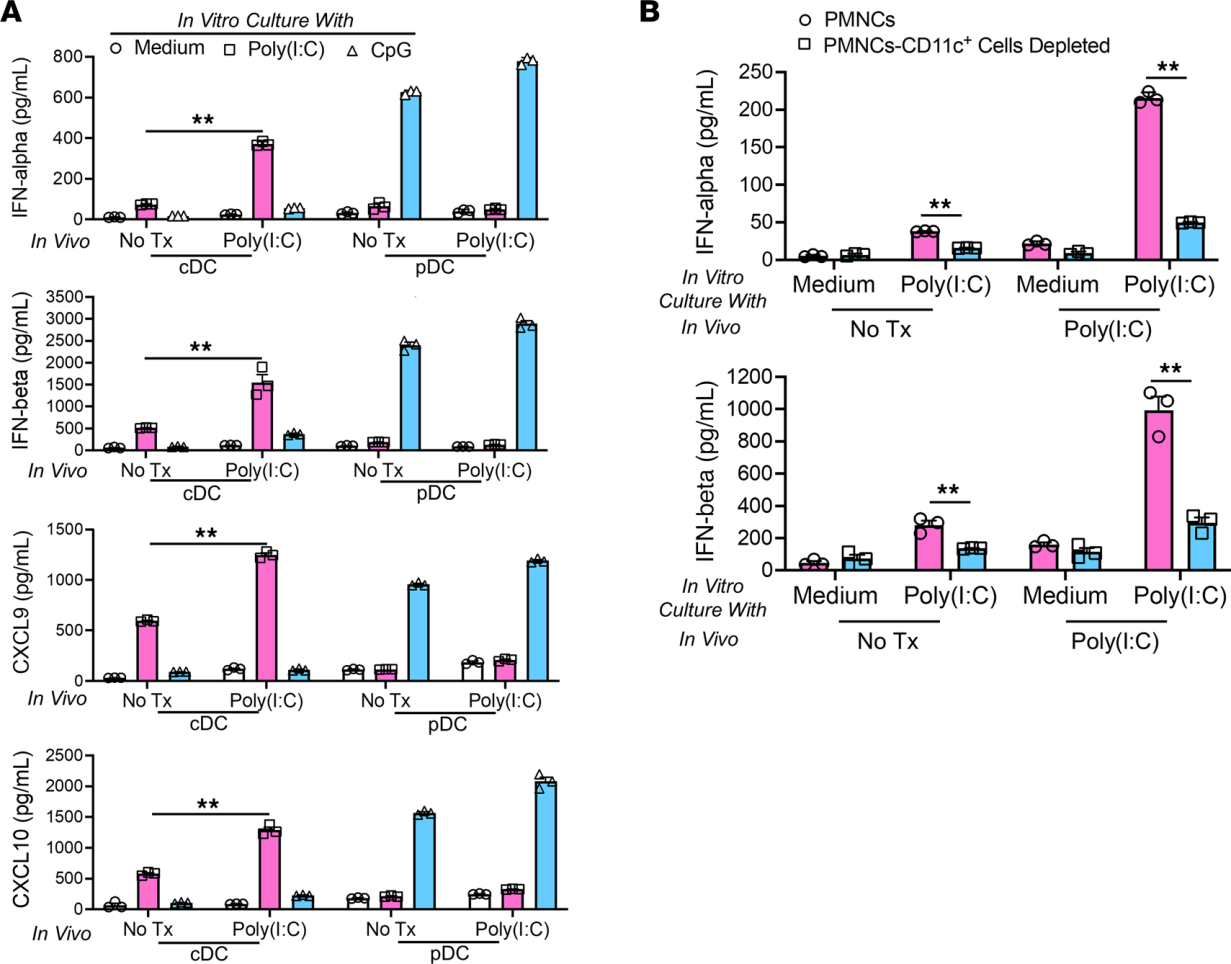
Conventional DCs are the major cellular source of type I IFNs in the inductive phase of experimental autoimmune pancreatitis. MRL/MpJ mice (*n* = 6) were treated with poly(I:C) by intraperitoneal injection for a total of 2 times. After sacrifice at 3 hours following the final injection, pancreases were removed and processed to extract pancreatic mononuclear cells (PMNCs). PMNCs were extracted from untreated MRL/MpJ mice (*n* = 6) to obtain baseline data (No Tx). (**A**) Conventional DCs (cDCs, 1 × 10^6^/mL) and pDCs (1 × 10^6^/mL) were purified from the extracted PMNCs and cultured with poly(I:C) (25 μg/mL) or CpG (1 μM) for 48 hours in triplicate. Culture supernatants from each well were then analyzed by ELISA to determine the concentrations of IFN-α, IFN-β, CXCL9, and CXCL10. (**B**) PMNCs (1 × 10^6^/mL) and CD11c^+^ DC–depleted PMNCs (1 × 10^6^/mL) were stimulated with poly(I:C) (25 μg/mL) for 48 hours in triplicate. Culture supernatant from each well was then analyzed by ELISA. Each dot represents the value derived from 1 well. Statistical analyses were performed using an unpaired, 2-tailed Student’s *t* test. Results shown are representative of data derived from 2 independent experiments and are expressed as mean ± SEM. **P < 0.01.

The above data strongly suggested that resident cDCs bearing TLR3 were the cells responding to poly(I:C) during the early induction phase of AIP. To verify that this was the case, we determined the responses of purified pancreatic cDCs and pDCs to poly(I:C) isolated from mice in the early phase of AIP induction (i.e., after administration of a second dose of poly[I:C]). We found that at this early time point the cDCs, but not the pDCs, produced large amounts of IFN-α, IFN-β, CXCL9, and CXCL10 upon stimulation with poly(I:C) (Figure 2A); pDCs could also produce these factors, but only when stimulated by CpG, a TLR9 ligand that was not the initiating driver of disease. Finally, it should be noted that the cDCs isolated from poly(I:C)-treated mice produced higher levels of type I IFNs and chemokines upon stimulation with poly(I:C) than cDCs from untreated mice, suggesting that cDCs present in the milieu characterizing the early phase of AIP have gained enhanced responsivity to TLR3 stimulation.

TLR3 expression is not limited to the CD11c^+^ cDCs (21). Therefore, in a final set of studies relating to TLR3 expression in early experimental AIP, we performed cell depletion studies to verify that cDCs were indeed the main source of type I IFNs at this stage of disease. Accordingly, PMNCs isolated from the mice after treatment with the second dose of poly(I:C) were depleted of CD11c^+^ cells by anti-CD11c MACS beads and then stimulated with poly(I:C). As previously observed, PMNCs isolated from poly(I:C)-treated mice produced greater amounts of IFN-α and IFN-β than those from untreated mice (Figure 2B); however, depletion of CD11c^+^ cells markedly reduced production of type I IFNs. Thus, pancreatic cDCs, at this early phase of AIP induction, are indeed the main producers of type I IFNs upon sensing of poly(I:C) by TLR3 and the possible contribution of other cell types to such production is small.

Taken together, these studies provide evidence that the development of experimental AIP following repeated poly(I:C) administration is initiated by TLR3^+^ cDCs producing type I IFNs, CXCL9, and CXCL10.

### The MRL/MpJ model of experimental AIP is marked by migration of CXCR3^+^ T cells into the pancreas.

**Figure 3:**
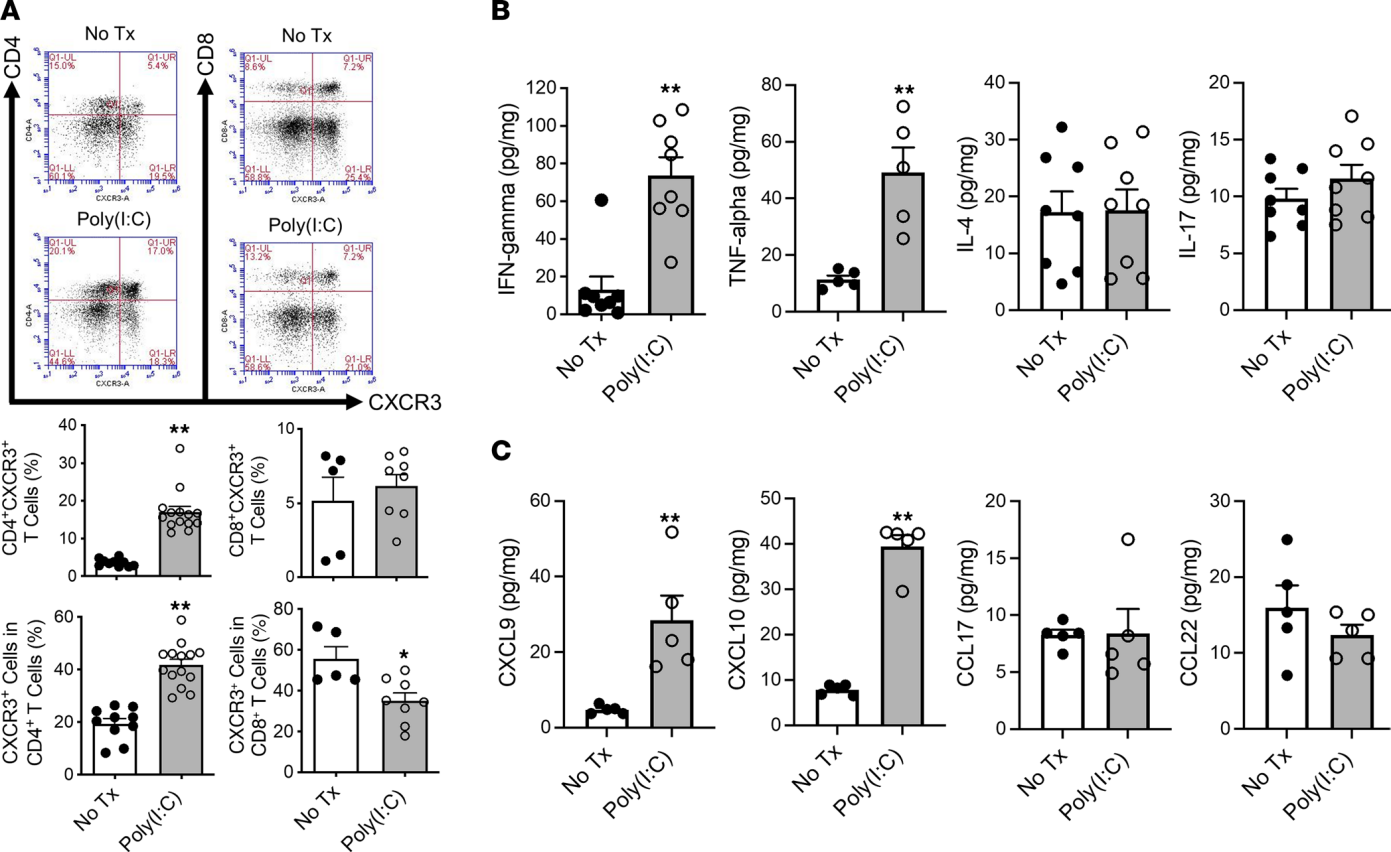
Expression of Th1-associated cytokines and chemokines is enhanced in MRL/MpJ mice treated with repeated injections of poly(I:C). MRL/MpJ mice (*n* = 14) were treated with poly(I:C) by intraperitoneal injection twice a week for a total of 16 times. After sacrifice at 3 hours following the final injection, pancreases were removed and processed to extract pancreatic mononuclear cells (PMNCs) and proteins. PMNCs and proteins were extracted from untreated MRL/MpJ mice (*n* = 10, No Tx) to obtain baseline data. (**A**) Representative flow cytometric analyses (with gates set on lymphocyte fraction, upper panel) and bar graphs of cumulative results (lower panel) showing the percentages of CD4^+^CXCR3^+^ T cells and CD8^+^CXCR3^+^ T cells among PMNCs obtained from individual mice. (**B** and **C**) Concentrations of IFN-γ, TNF-α, IL-4, IL-17, CXCL9, CXCL10, CCL17, and CCL22 in protein extracts of pancreatic tissues from mice. Each dot represents the value derived from 1 mouse. Statistical analyses were performed using an unpaired, 2-tailed Student’s *t* test. Results shown represent the combined data of 3 (**A**) or 2 (**B**) independent experiments. Results are expressed as mean ± SEM. *P < 0.05, **P < 0.01.

Recognizing that poly(I:C) activation of TLR3 in cDCs residing in the uninflamed pancreas and then in the nascent inflamed pancreas would induce production of type I IFNs and Th1 chemokines (CXCL9, CXCL10), we reasoned that poly(I:C) induction of AIP could be accompanied by an early downstream Th1 cell–oriented cytokine and chemokine response that could lead to induction of Th1 cells in lymph nodes draining the pancreas and their migration into the pancreas. In studies addressing this possibility, we found that the percentage of pancreatic CD4^+^CXCR3^+^ T cells (i.e., Th1 cells) among PMNCs in MRL/MpJ mice administered poly(I:C) 16 times was much greater than those of untreated mice, whereas in contrast, such treatment did not increase the percentage of CD8^+^CXCR3^+^ T cells (Figure 3A) and caused a slight decrease in the percentage of CCR4^+^ cells within the CD3^+^ T cell population (i.e., Th2 cells) (Supplemental Figure 2C). As expected from the presence of CXCR3^+^ Th1 cells, we found that the levels of IFN-γ and TNF-α were greater in the pancreas of poly(I:C)-treated mice than in the pancreas of untreated mice, whereas no significant difference was seen in the levels of IL-4 or IL-17 in these groups of mice (Figure 3B). In addition, pancreatic expression of CXCL9 and CXCL10 (i.e., chemokines targeting CXCR3 on Th1 cells; ref. 20) was markedly enhanced in treated mice as compared with those in untreated mice, whereas in contrast, pancreatic expression of CCL17 and CCL22, chemokines targeting CCR4 expressed on Th2 cells (20), was comparable in mice with and without poly(I:C) treatment (Figure 3C). Finally, since CD4^+^ cytotoxic T lymphocytes (CTLs) expressing SLAMF7, granzyme A, or granzyme B are implicated in the immunopathogenesis of human IgG4-RD (9, 17), we determined whether the CD4^+^CXCR3^+^ T cells isolated from the pancreas of mice administered poly(I:C) express SLAMF7 or granzyme B by cell surface or intracellular flow cytometric staining, respectively, but found no such staining (Supplemental Figure 3A). Thus, it is unlikely that CD4^+^ CTLs play a major role in the pathogenesis of experimental AIP at this stage of the disease.

The above characterization of pancreatic CD4^+^CXCR3^+^ T cells was performed on PMNCs isolated from MRL/MpJ mice after a full poly(I:C) 16-dose administration regimen when the pancreas was already heavily populated by pDCs. It was therefore of interest to determine whether a similar T cell population was present in the pancreas at an earlier time point when, as shown above, pancreatic cDCs rather than pDCs were still the main source of type I IFNs and chemokines attracting CD4^+^CXCR3^+^ T cells into the pancreas. To explore this question, we compared accumulation of CD4^+^CXCR3^+^ T cells as assessed by percentage of CD4^+^CXCR3^+^ T cells among PMNCs after administration of 3 doses of poly(I:C) with that after administration of 16 doses of poly(I:C). We found that whereas the percentage of pDCs was only mildly increased after 3 doses, the percentage of CD4^+^CXCR3^+^ T cells after 3 doses was equal to that after 16 doses (Supplemental Figure 3B). This early accumulation of CD4^+^CXCR3^+^ T cells is fully consistent with our hypothesis that initial stimulation of pancreatic TLR3-bearing cDCs by poly(I:C) results in IFN-α/β production and that the latter, in turn, leads to induction of Th1 responses (most likely in lymph nodes draining the pancreas) followed by early migration of CXCR3^+^ T cells into the pancreas via CXCL9 and CXCL10 secretion.

### The development of experimental AIP depends on the interaction between CXCL9 or CXCL10 and CXCR3.

**Figure 4:**
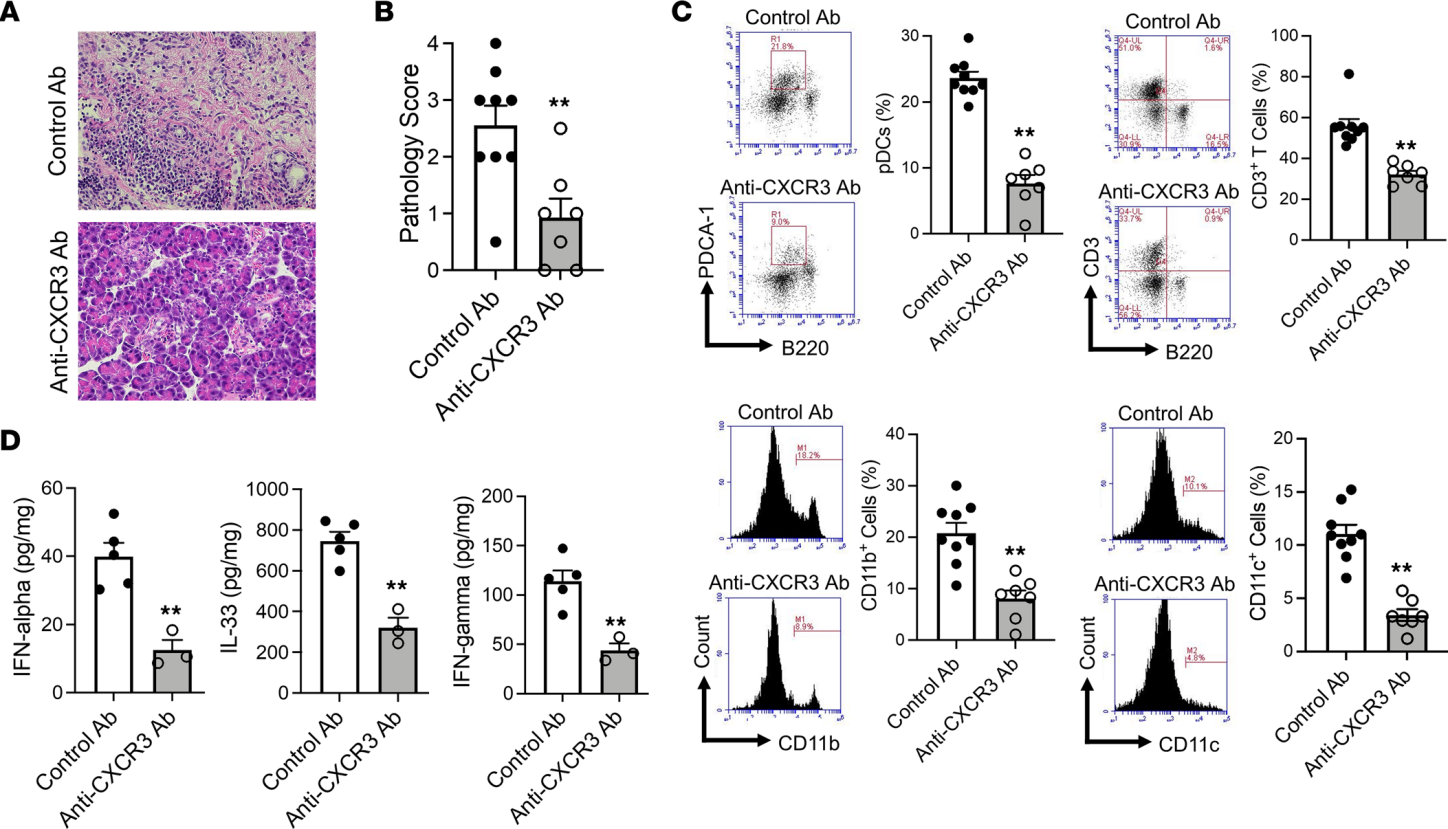
The development of experimental autoimmune pancreatitis is dependent on the CXCR3-mediated signaling pathways. MRL/MpJ mice were administered poly(I:C) twice a week for a total of 16 times by intraperitoneal injection. Each poly(I:C) injection was preceded by intraperitoneal injection of anti-CXCR3 Ab (200 μg, *n* = 7) or control Ab (200 μg, *n* = 9). After sacrifice at 3 hours following the final set of injections, pancreases were removed and analyzed as indicated. (**A** and **B**) Representative hematoxylin and eosin staining of the pancreatic tissues and pathological scores for autoimmune pancreatitis of individual mice in the 2 groups. Original magnification, ×400. (**C**) Flow cytometric analyses showing the percentages of pDCs, CD3^+^ T cells, CD11b^+^ myeloid cells, and CD11c^+^ DCs among pancreatic mononuclear cells. pDCs were defined as PDCA-1^+^B220^lo^ cells. Left panels: Representative flow cytometric analyses. Right panels: Bar graphs of cumulative data from individual mice. (**D**) Concentrations of IFN-α, IL-33, and IFN-γ in protein extracts of pancreatic tissues obtained from mice as determined by ELISA. Statistical analyses were performed using an unpaired, 2-tailed Student’s *t* test. The results shown are the combined data derived from 2 independent experiments. Each dot represents the value derived from 1 mouse. Results are expressed as mean ± SEM. **P < 0.01.

The above data showing that CXCL9 and/or CXCL10 expression was associated with accumulation of CXCR3^+^ T cells in the inflamed pancreas of mice with experimental AIP raised the question of whether this CXCL9/CXCL10-CXCR3 interaction is required for the development of AIP. To answer this question, we determined the effect of blocking CXCL9/CXCL10-CXCR3 interaction on development of AIP by administration of a neutralizing antibody (Ab) against CXCR3 or control Ab at the time of each poly(I:C) injection. We found that, as first evaluated by AIP scores, such blockade prevented the development of experimental AIP (Figure 4, A and B). Moreover, this attenuation of AIP was accompanied by a reduction in the pancreatic accumulation of pDCs as well as CD3^+^ T cells; in addition, it was accompanied by the reduced percentages of CD11b^+^ myeloid cells and CD11c^+^ DCs with reduced CD11b and CD11c expression, as determined by MFI (Figure 4C and Supplemental Figure 2B). Finally, consistent with the reduction in pancreatic pDCs, pancreatic expression of IFN-α, IL-33, and IFN-γ was also markedly decreased in the anti-CXCR3 Ab plus poly(I:C)–treated mice as compared with those treated with control Ab plus poly(I:C) (Figure 4D). These data strongly suggest that the chemoattraction and accompanying migration of CXCR3^+^ T cells into the pancreas mediated by CXCL9 and/or CXCL10 is necessary for the development of experimental AIP. In addition, since such migration was required for proinflammatory cytokine production, these data also imply that the interaction between CD4^+^CXCR3^+^ T cells and CD11c^+^ DCs during the induction phase of AIP is indispensable for the subsequent development of mature AIP. Parenthetically, the dependence of the pancreatitis on these chemokine ligand-receptor interactions also implies that Th1 cell development is occurring outside of the pancreas.

### Pancreatic accumulation of pDCs requires interaction between CCR9 and CCL25.

As indicated above, the initial source of type I IFNs mediating pancreatic migration of CXCR3^+^ T cells is likely to be TLR3-bearing pancreatic cDCs susceptible to stimulation by poly(I:C) rather than pancreatic pDCs, since the latter cells express low levels of TLR3.

However, the development of AIP/IgG4-RD is accompanied by a huge increase in the number of pancreatic pDCs and the latter cells (not cDCs) are the source of IFN-α in the mature inflammation. Indeed, the kinetic studies described above clearly showed that the percentages of pancreatic pDCs were much higher in mice treated with 16 doses of poly(I:C) than in those treated with zero or 3 doses (Supplemental Figure 3B). This raised the question of the mechanism underlying pDC accumulation in the nascent inflamed pancreas. Accordingly, we next investigated the chemokine interactions that could facilitate pDC migration into the pancreas in the next stage of developing experimental AIP.

**Figure 5:**
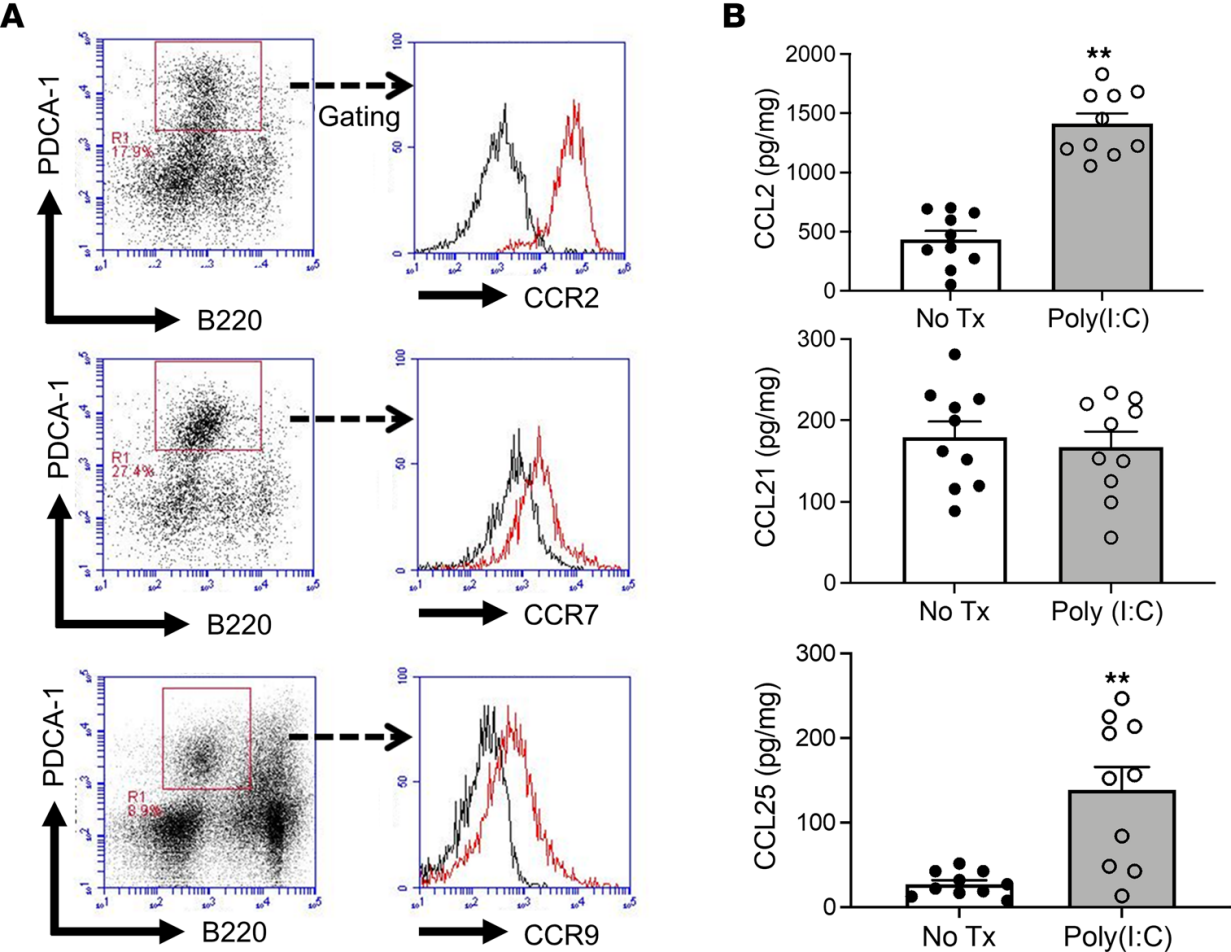
Expression of CCR9 on pDCs in MRL/MpJ mice treated with repeated injections of poly(I:C). MRL/MpJ mice were treated with or without poly(I:C), as indicated in Figure 3. After sacrifice at 3 hours following the final set of injections, pancreases were removed and analyzed as indicated. (**A**) Flow cytometric analysis of cell membrane expression of CCR2, CCR7, and CCR9 on pDCs in pancreatic mononuclear cells extracted from MRL/MpJ mice treated with 16 doses of poly(I:C). Analysis gate was set on PDCA-1^+^B220^lo^ pDCs. Black curve: control Ab. Red curve: CCR2, CCR7, and CCR9 Abs. Results shown are representative data from 3 mice. (**B**) Concentrations of CCL2, CCL21, and CCL25 in protein extracts of pancreatic tissues obtained from untreated mice (No Tx, *n* = 10) or mice treated with 16 injections of poly(I:C) (*n* = 10), as determined by ELISA. Each dot represents the value derived from 1 mouse. Statistical analyses were performed using an unpaired, 2-tailed Student’s *t* test. Results shown are combined data of 2 independent experiments (**B**). Results are expressed as mean ± SEM. **P < 0.01.

In initial studies, we determined that pancreatic pDCs isolated from mice with poly(I:C)-induced AIP, as do pDCs generally, express CCR2, CCR7, and CCR9 (22) (Figure 5A). We therefore next assessed pancreatic expression of chemokine ligands that bind to and attract pDCs bearing these receptors, CCL2, CCL21, and CCL25 (23–25). We found that following administration of repeated doses of poly(I:C), pancreatic tissue expressed increased levels of CCL2 and CCL25, but not CCL21 (Figure 5B), suggesting that pDCs utilize molecular interactions between CCR2 and CCL2 or between CCR9 and CCL25 to migrate to the pancreas.

**Figure 6:**
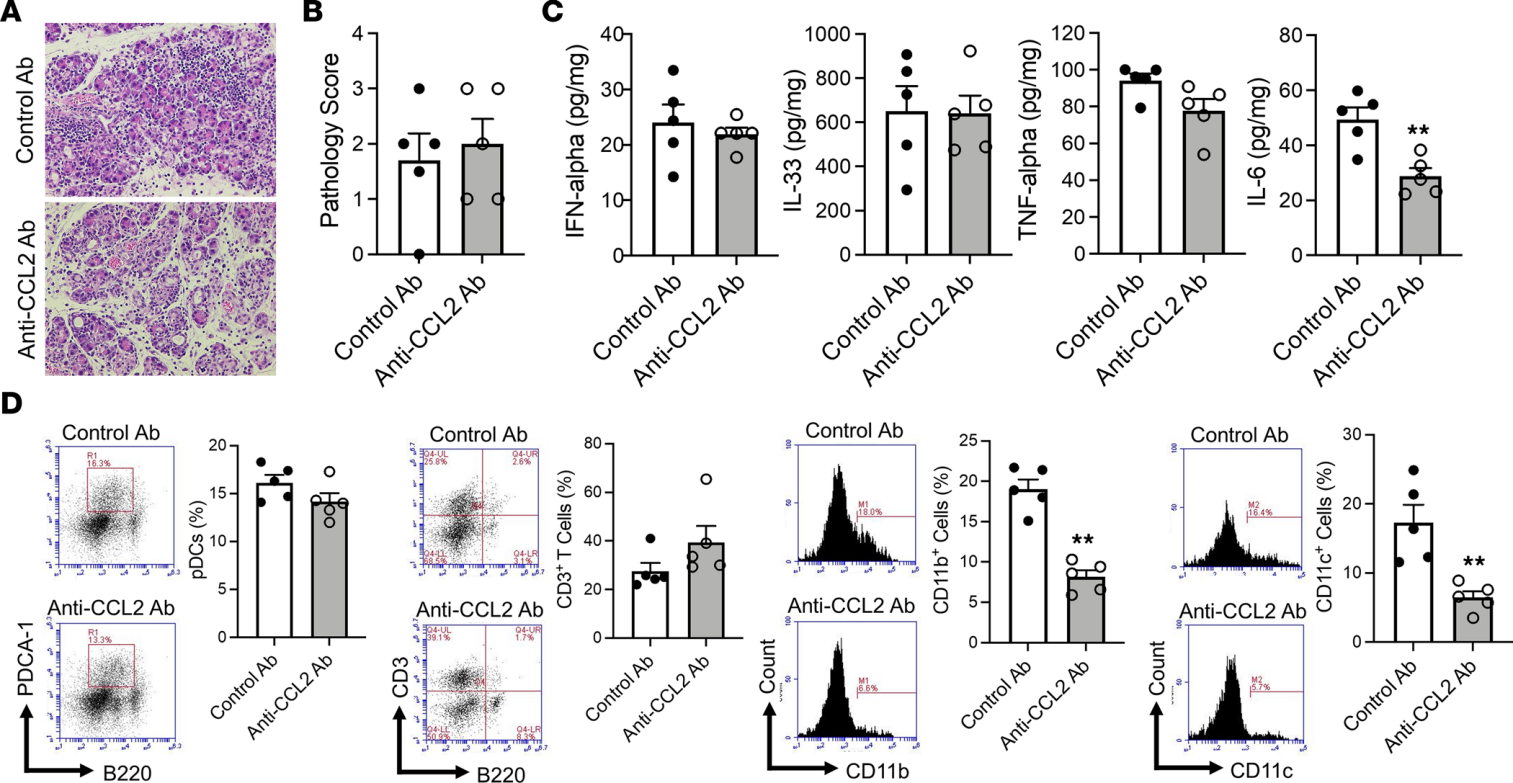
Pancreatic accumulation of pDCs does not require the molecular interaction between CCR2 and CCL2. MRL/MpJ mice were administered poly(I:C) twice a week for a total of 16 times. Each poly(I:C) injection was preceded by intraperitoneal injection of anti-CCL2 Ab (200 μg, *n* = 5) or control Ab (200 μg, *n* = 5). After sacrifice at 3 hours following the final set of injections, pancreases were removed and analyzed as indicated. (**A** and **B**) Representative hematoxylin and eosin staining of the pancreatic tissues and pathological scores of induced autoimmune pancreatitis in the 2 groups. Original magnification, ×400. (**C**) Concentrations of IFN-α, IL-33, TNF-α, and IL-6 in protein extracts of pancreatic tissues obtained from mice in the 2 groups. (**D**) Flow cytometric analyses showing the percentages of pDCs, CD3^+^ T cells, CD11b^+^ myeloid cells, and CD11c^+^ DCs among pancreatic mononuclear cells. Left panels: Representative flow cytometric analyses. Right panels: Bar graphs of cumulative data from individual mice. pDCs were defined as PDCA-1^+^B220^lo^ cells. Each dot represents the value derived from 1 mouse. Statistical analyses were performed using an unpaired, 2-tailed Student’s *t* test. Results are expressed as mean ± SEM. **P < 0.01.

To determine whether these interactions were in fact necessary for the development of pancreatitis, we first assessed the effect of neutralizing Ab against CCL2 or CCL25 administered at the time of poly(I:C) administration. We found that the development of AIP, as measured by pathological scores, was comparable between mice treated with control Ab or with anti-CCL2 Ab (Figure 6, A and B). Likewise, administration of this Ab was not accompanied by significant changes in pancreatic expression of IFN-α, IL-33, or TNF-α (Figure 6C). Finally, administration of anti-CCL2 Ab did not reduce the percentage of pDCs as compared with administration of control Ab, although such treatment reduced the percentages of CD11b^+^ myeloid cells and CD11c^+^ DCs and tended to reduce their expression of CD11b and CD11c (Figure 6D and Supplemental Figure 2B). It should be noted that since the reduction in CD11c^+^ DC percentage by anti-CCL2 Ab administration is likely to be a consequence of cell migration, it reflects the lack of endogenous expansion of the initially present resident CD11c^+^ DC population during the development of pancreatitis; thus, the reduction in CD11c^+^ DCs suggests that this cell population is dispensable when the pancreatic inflammation has reached a mature stage.

**Figure 7:**
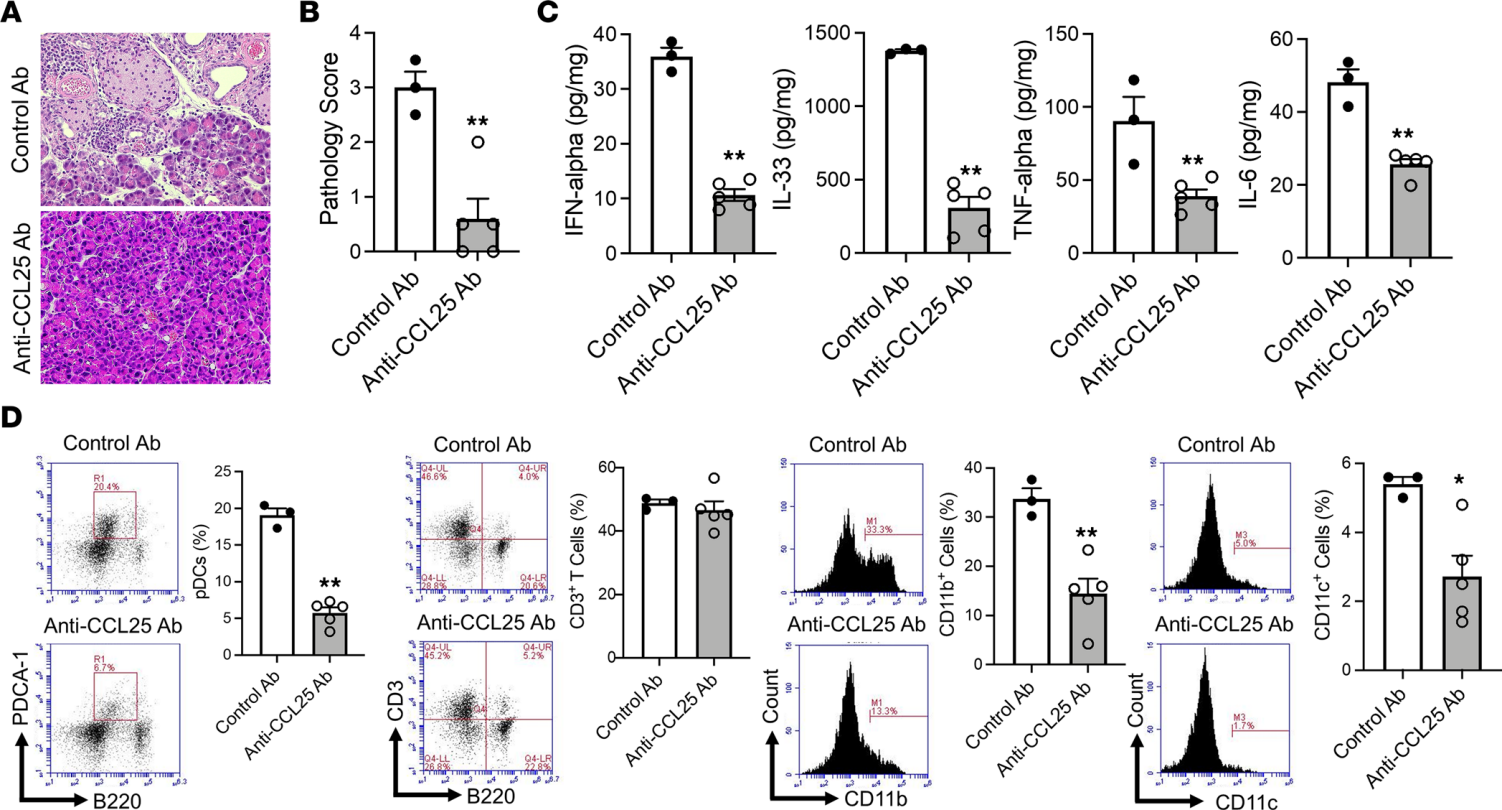
Pancreatic accumulation of pDCs requires the molecular interaction between CCR9 and CCL25. MRL/MpJ mice were administered poly(I:C) twice a week for a total of 16 times by intraperitoneal injection. Each poly(I:C) injection was preceded by intraperitoneal injection of anti-CCL25 Ab (100 μg, *n* = 5) or control Ab (100 μg, *n* = 3). After sacrifice at 3 hours following the final set of injections, pancreases were removed and analyzed as indicated. (**A** and **B**) Representative hematoxylin and eosin staining of the pancreatic tissues and pathological scores of induced autoimmune pancreatitis in the 2 groups. Original magnification, ×400. (**C**) Concentrations of IFN-α, IL-33, TNF-α, and IL-6 in protein extracts of pancreatic tissues obtained from mice in the 2 groups. (**D**) Flow cytometric analyses showing the percentages of pDCs, CD3^+^ T cells, CD11b^+^ myeloid cells, and CD11c^+^ DCs among pancreatic mononuclear cells. Left panels: Representative flow cytometric analyses. Right panels: Bar graphs of cumulative data from individual mice. pDCs were defined as PDCA-1^+^B220^lo^ cells. Each dot represents the value derived from 1 mouse. Statistical analyses were performed using an unpaired, 2-tailed Student’s *t* test. Results are expressed as mean ± SEM. *P < 0.05, **P < 0.01.

In parallel studies, we investigated the involvement of the interaction between CCR9 and CCL25 by assessment of pancreatitis following administration of anti-CCL25 Ab or control Ab. In this case, we found that, as determined by pathological scores, the blockade of the CCR9-CCL25 interaction by anti-CCL25 Ab markedly inhibited the development of experimental AIP (Figure 7, A and B). In addition, pancreatic expression of IFN-α, IL-33, TNF-α, and IL-6 was markedly decreased following administration of anti-CCL25 Ab as compared with administration of control Ab (Figure 7C). Finally, consistent with these changes in cytokine profiles, treatment with anti-CCL25 Ab significantly reduced the percentages of pDCs, CD11b^+^ myeloid cells, and CD11c^+^ DCs as compared with the administration of control Ab; in addition, such treatment was accompanied by CD11b^+^ myeloid cells and CD11c^+^ DCs that tended to have reduced CD11b and CD11c expression, respectively (Figure 7D and Supplemental Figure 2B). Thus, these data suggest that pDC migration is necessary for the development of experimental AIP and that such migration is mediated by interaction between CCL9 and CCL25, but not that between CCR2 and CCL2.

### Pancreatic CD3^+^ T cells stimulated by IFN-α are the source of CCL25 production.

**Figure 8:**
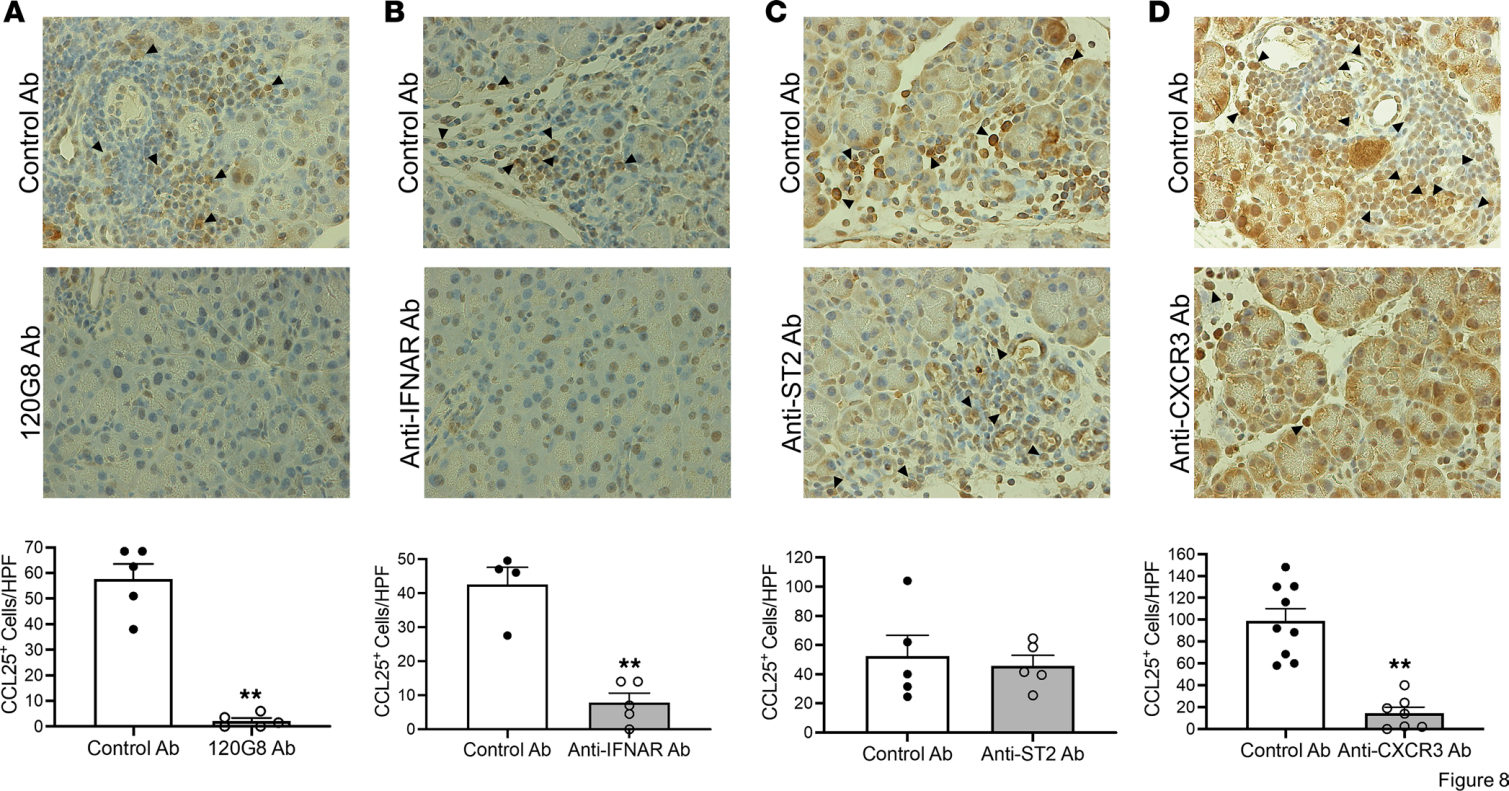
Pancreatic lymphoid cells stimulated by IFN-α are the source of CCL25. MRL/MpJ mice were administered poly(I:C) twice a week for a total of 16 times by intraperitoneal injection. Each poly(I:C) injection was preceded by intraperitoneal injection of (**A**) pDC-depleting Ab (120G8 Ab, 100 μg, *n* = 5) or control Ab (100 μg, *n* = 5); (**B**) anti–IFN-α/β receptor Ab (anti-IFNAR Ab, 100 μg, *n* = 5) or control Ab (100 μg, *n* = 4); (**C**) anti–IL-33 receptor Ab (anti-ST2 Ab, 100 μg, *n* = 5) or control Ab (100 μg, *n* = 5); and (**D**) anti-CXCR3 Ab (200 μg, *n* = 7) or control Ab (200 μg, *n* = 9). After sacrifice at 3 hours following the final set of injections, pancreases were removed and processed for immunohistochemical studies with anti-CCL25 Ab. Upper panels: Representative tissue images of CCL25 expression by morphologically identified lymphoid cells (indicated by arrowheads) in tissues obtained from mice administered control Ab or depleting/neutralizing Ab. Original magnification, ×800. Lower panels: Bar graphs showing numbers of CCL25^+^ lymphoid cells/high-powered field (HPF) (counted blindly). Each dot represents the value derived from 1 mouse. Statistical analyses were performed using an unpaired, 2-tailed Student’s *t* test. Results are expressed as mean ± SEM. **P < 0.01.

Having obtained evidence that interaction between CCR9 and CCL25 plays an important role in the migration of pDCs into the pancreas, we conducted studies to identify the cellular source of CCL25 and the cytokines inducing its secretion. Here, we first performed an immunofluorescence analysis of inflamed pancreatic tissue from mice administered repeated 16 doses of poly(I:C) in the usual AIP induction regimen and found that cells bearing surface CD3 (i.e., CD3^+^ T cells) express intracellular CCL25 (Supplemental Figure 3C), whereas amylase-positive cells (pancreatic acinar cells) do not express CCL25 (A Hara, unpublished observation). We then determined the cell and cytokine environment supporting such CD3^+^ T cell CCL25 production. For this purpose, we examined pancreatic tissues from mice administered repeated 16 doses of poly(I:C) in the usual AIP induction regimen, but in this case in combination with anti-pDC Ab (120G8 Ab), anti–IFN-α/β receptor Ab (anti-IFNAR Ab), anti-CXCR3 Ab, and anti–IL-33 receptor Ab (anti-ST2 Ab) and then evaluated the expression of CCL25 in pancreatic tissues by blindly counting immunoreactive CCL25^+^ cells that were morphologically identified as lymphoid cells (4, 5). We found that the number of such cells positive for CCL25 was markedly decreased by treatment with 120G8 Ab, anti-IFNAR Ab, or anti-CXCR3 Ab, but not decreased by treatment with anti-ST2 Ab upon the blinded study (Figure 8, A–D). Thus, CD3^+^ T cells bearing CXCR3 and stimulated by IFN-α, but not IL-33, are the source of CCL25 production.

### Pathogenic pDCs in the inflamed pancreas produce high levels of IFN-α when stimulated by CXCR3^+^ T cells producing CCL25.

**Figure 9:**
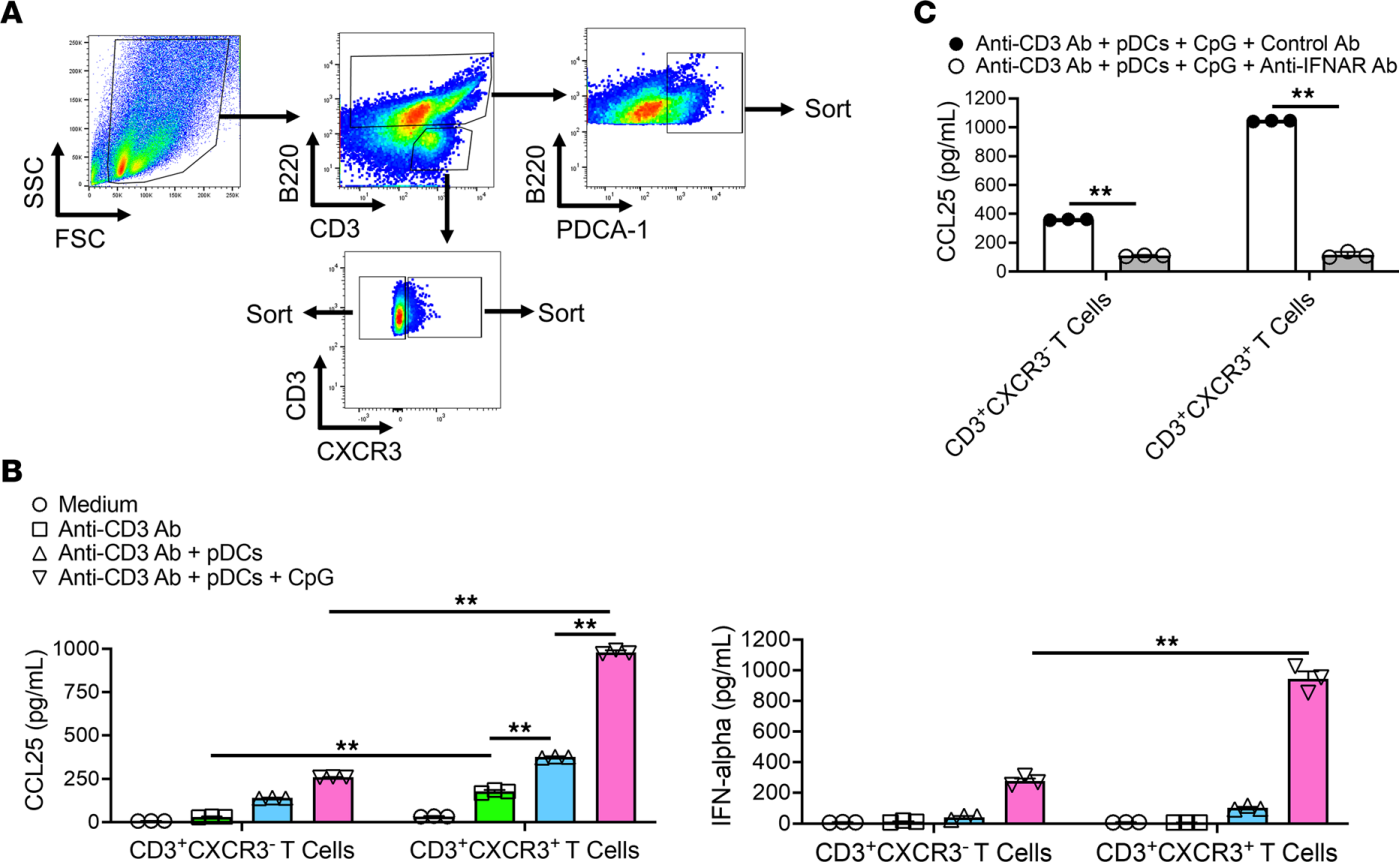
CD3^+^CXCR3^+^ T cells produce CCL25 in response to IFN-α secreted by pDCs. (**A**) FACS gating strategy for acquisition of CD3^+^CXCR3^+^ T cells, CD3^+^CXCR3^–^ T cells, and PDCA-1^+^B220^lo^ pDCs. (**B** and **C**) Pancreatic mononuclear cells (PMNCs) were isolated from pancreases of MRL/MpJ mice (*n* = 3) administered poly(I:C) twice a week for a total of 16 times. PMNCs were subjected to FACS to acquire purified CD3^+^CXCR3^+^ T cells, CD3^+^CXCR3^–^ T cells, and pDCs, after which they were cultured or cocultured for 48 hours (at 1 × 10^5^/mL) in triplicate in the presence of anti-CD3 Ab (5 μg/mL) alone or with CpG (1 μM); in addition, in some experiments, anti–IFN-α/β receptor Ab (anti-IFNAR Ab, 100 μg/mL) or control Ab was added to the culture. Cell culture supernatants from each well were analyzed by ELISA for measurement of CCL25 and IFN-α. Each dot represents the CCL25 or IFN-α concentration value derived from each well. Statistical analyses were performed using the Kruskal-Wallis test and Bonferroni-corrected Mann-Whitney *U* test (**B**) or an unpaired, 2-tailed Student’s *t* test (**C**). Results shown are the representative 1 of 2 independent experiments and are expressed as mean ± SEM. **P < 0.01.

Whereas the initial source of IFN-α leading to influx of CCL25-producing T cells are TLR3^+^ cDCs responding to poly(I:C), once T cells are attracted into the inflamed pancreatic tissue and draw pDCs into this tissue, it is likely that the latter cells become the chief source of IFN-α. To examine this possibility, we first conducted in vitro studies utilizing flow cytometry–sorted CD3^+^CXCR3^+^ T cells, CD3^+^CXCR3^–^ T cells, and pDCs obtained from inflamed pancreatic tissues of mice administered 16 doses of poly(I:C) to examine production of CCL25 and IFN-α within the mature inflamed pancreas (Figure 9A). We found that anti-CD3 Ab–stimulated CD3^+^CXCR3^+^ T cells secreted significantly higher levels of CCL25 than anti-CD3 Ab–stimulated CD3^+^CXCR3^-^ T cells and in both cases, such secretion was greatly augmented by the presence of pDCs, particularly when the latter cells were stimulated by TLR9 ligand (CpG) (Figure 9B). In addition, pDC augmentation was substantially blocked by anti-IFNAR Ab, indicating that this effect was dependent on IFN-α production by pDCs (Figure 9, B and C). In line with these findings, CpG-stimulated pDCs secreted large amounts of IFN-α when cocultured with CD3^+^CXCR3^+^ T cells, but not CD3^+^CXCR3^–^ T cells (Figure 9B). Thus, these in vitro studies show that CD3^+^CXCR3^+^ T cells are the source of high-level CCL25 secretion in the inflamed pancreas and that such secretion is dependent on pDC secretion of IFN-α.

We next conducted in vitro studies utilizing flow cytometry–sorted CD3^+^CXCR3^+^ T cells, CD3^+^CXCR3^–^ T cells, and cDCs obtained from pancreatic tissues of mice administered 3 poly(I:C) doses to examine CD3^+^ T cell production of CCL25 during the induction phase of experimental AIP (Supplemental Figure 4A). We found that CD3^+^CXCR3^+^ T cells produced greater amounts of CCL25 than CD3^+^CXCR3^–^ T cells upon stimulation with anti-CD3 Ab in the presence of cDCs (Supplemental Figure 4B). In addition, the CCL25 production by CD3^+^CXCR3^+^ T cells was dependent on IFN-α since the addition of anti-IFNAR Ab reduced CCL25 production (Supplemental Figure 4B). It is worth noting that the amounts of CCL25 derived from CD3^+^CXCR3^+^ T cells in the presence of cDCs were smaller than those from the same T cell population in the presence of pDCs (Figure 9B and Supplemental Figure 4B).

**Figure 10:**
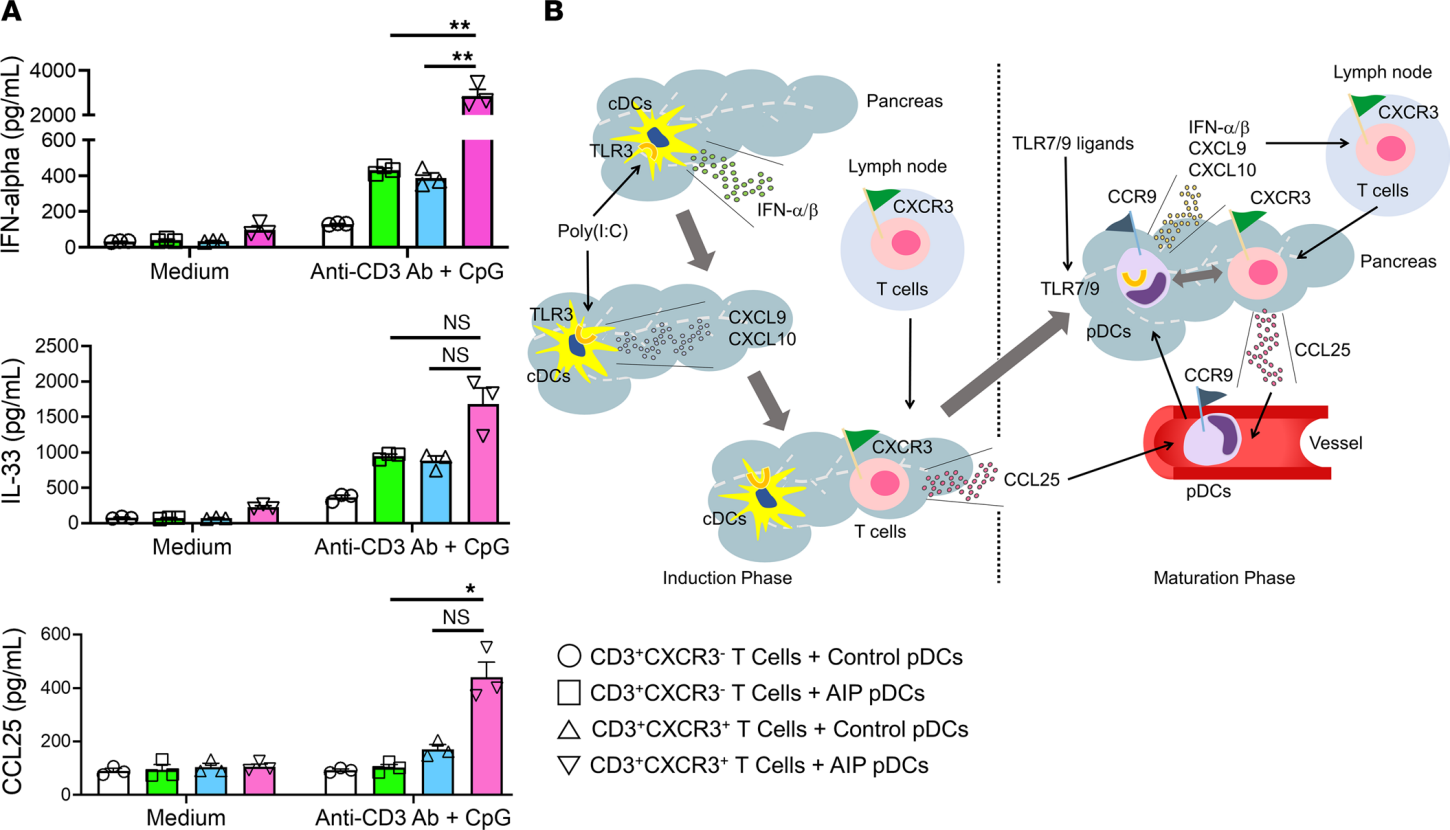
CD3^+^CXCR3^+^ T cells enhance differentiation of pDCs producing IFN-α and IL-33. (**A**) Purified CD3^+^CXCR3^+^ T cells, CD3^+^CXCR3^–^ T cells, and pDCs were acquired from the pancreatic tissues of MRL/MpJ mice administered poly(I:C) (*n* = 3) or untreated MRL/MpJ mice (*n* = 3) and cocultured as described in Figure 9 in triplicate under the indicated conditions. Cell culture supernatants from each well were analyzed by ELISA for measurement of CCL25, IFN-α, and IL-33. Each dot represents the value derived from 1 well. Statistical analyses were performed using the Kruskal-Wallis test and Bonferroni-corrected Mann-Whitney *U* test. Results are expressed as mean ± SEM. *P < 0.05, **P < 0.01. NS, not significant. (**B**) Diagram illustrating the positive feedback loop consisting of IFN-α/β, CXCL9, CXCL10, and CCL25 that establishes and sustains experimental pDC-driven autoimmune pancreatitis (see text for full description).

In a final set of studies along these lines, we examined whether pDC production of IFN-α and IL-33 was influenced by interaction with activated T cells within the inflamed pancreas. To this end, pDCs from the pancreas of nontreated and poly(I:C)-treated MRL/MpJ mice and CD3^+^CXCR3^+^ T cells and CD3^+^CXCR3^–^ T cells from the pancreas of poly(I:C)-treated MRL/MpJ mice were isolated by flow cytometry and then cocultured as indicated in Figure 9 in the presence or absence of anti-CD3 Ab or CpG. We found that pDCs isolated from the inflamed pancreas of mice secreted greater amounts of IFN-α and IL-33 than pDCs isolated from the uninflamed pancreas of mice when cultured in the presence of CpG (Figure 10A). In addition, pDCs isolated from inflamed pancreas, but not uninflamed pancreas, exhibited greatly increased IFN-α secretion and substantially increased IL-33 secretion when cocultured with activated CD3^+^CXCR3^+^ T cells rather than with CD3^+^CXCR3^–^ T cells (Figure 10A). Finally, production of CCL25 was enhanced in a coculture composed of CD3^+^CXCR3^+^ T cells and pDCs isolated from the pancreas displaying established AIP (Figure 10A). These findings suggest that residency in the inflamed pancreas induces changes in the pDCs that allow enhanced costimulation by CD3^+^CXCR3^+^ T cells and greater responsiveness to TLR9 stimulation. Such enhanced costimulation may consist in an increased responsiveness to CCL25 that is being produced by cross-stimulated (IFN-α–stimulated) CD3^+^CXCR3^+^ T cells by the activated pDCs (Figure 9C and Figure 10A).

Taken together, these data suggest first that TLR9 ligand–stimulated pDCs in the inflamed pancreas are remarkably good producers of both IFN-α and IL-33 when present in a milieu containing CD3^+^CXCR3^+^ T cells. Second, they suggest that pDCs in the inflamed pancreas have a heightened capacity to respond to CpG following interaction with CD3^+^CXCR3^+^ T cells secreting CCL25. These findings, along with the other findings discussed above, allow us to define a cytokine/chemokine cascade or positive feedback loop that establishes the cellular elements necessary for pancreatic inflammation in the MRL/MpJ model of AIP (Figure 10B). This feedback loop is initiated by sensing of poly(I:C) by TLR3-bearing cDCs that induce differentiation of Th1 cells in draining lymphoid tissues and pancreatic expression of CXCL9 and CXCL10, chemokines that mediate migration of induced CXCR3^+^ Th1 cells into the pancreas. Pancreatic CXCR3^+^ T cells then produce CCL25 in response to further stimulation by IFN-α that, in turn, induces recruitment of CCR9^+^ pDCs into the pancreas. Finally, the pDCs now resident in the pancreas and costimulated by CXCR3^+^ T cells as well as TLR7/9 ligands become the enduring source of IFN-α that reinitiates a T cell–CCL25-pDC–IFN-α loop capable of sustaining AIP/IgG4-RD.

### AIP/IgG4-RD in humans is marked by enhanced levels of serum CXCL9, CXCL10, and CCL25 that correlate with extent of disease.

**Figure 11:**
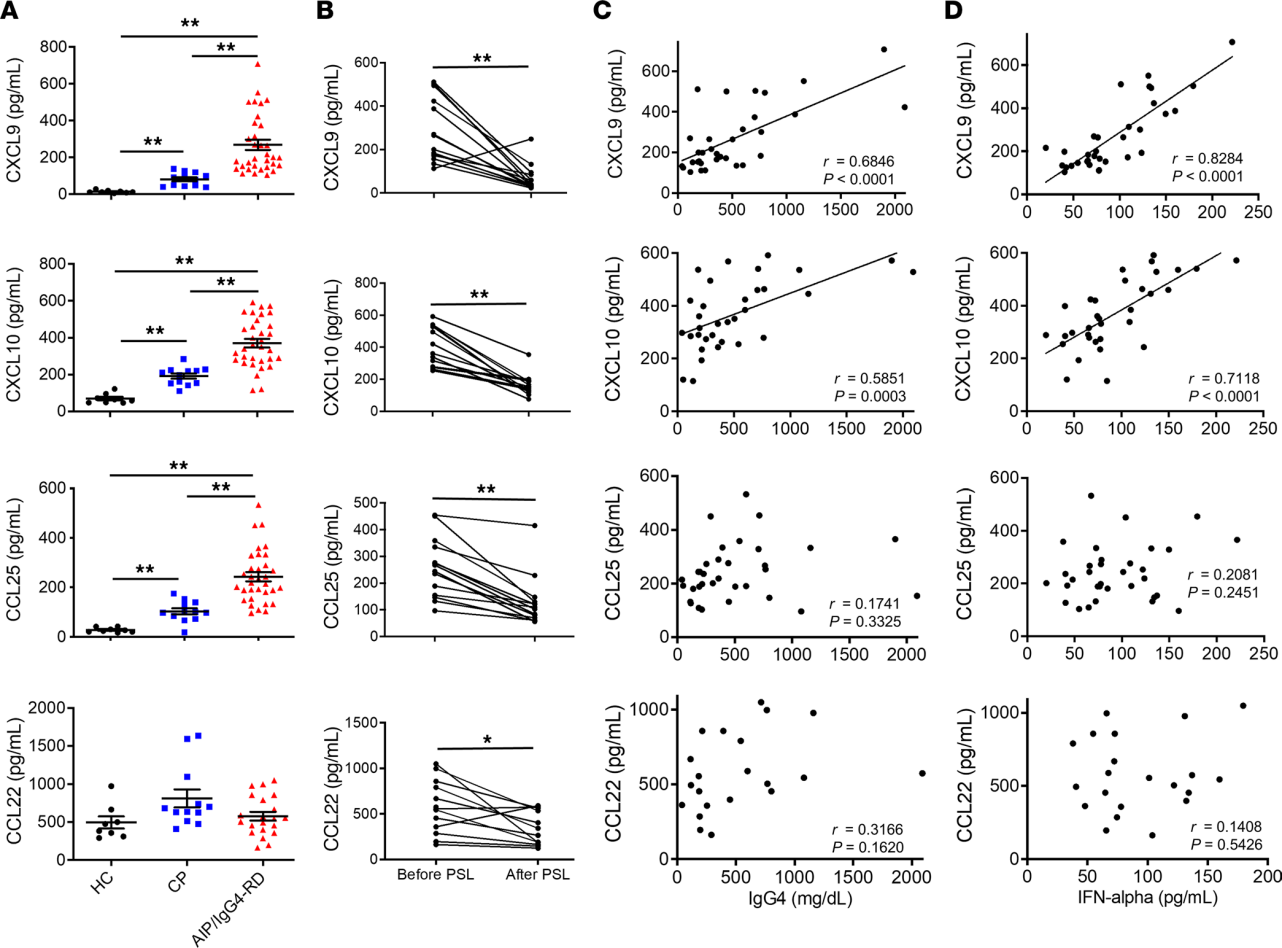
Serum concentrations of CXCL9, CXCL10, CCL25, and CCL22 in patients with autoimmune pancreatitis/IgG4-related disease. Serum samples were collected from healthy controls (HC, *n* = 8), chronic alcoholic pancreatitis (CP) patients (*n* = 12), and autoimmune pancreatitis (AIP)/IgG4-related disease (IgG4-RD) patients (*n* = 33). (**A**) Serum concentrations of CXCL9, CXCL10, CCL25, and CCL22; each dot corresponds to a value in 1 patient. Statistical analyses: Kruskal-Wallis test and Bonferroni-corrected Mann-Whitney *U* test. Results are expressed as mean ± SEM. (**B**) Serum chemokine levels from patients with AIP/IgG4-RD (*n* = 14) before and after induction of remission with prednisolone (PSL) therapy. Statistical analyses: Wilcoxon’s signed-rank test. (**C** and **D**) Correlation between serum IgG4 or IFN-α levels and chemokines in patients with AIP/IgG4-RD. Each dot represents 1 patient. *P* values and correlation coefficient (*r*) values, as determined by Spearman’s rank correlation test, are shown. *P < 0.05; **P < 0.01.

In the final series of the experiments, we investigated the question of whether the chemokines comprising the positive feedback loop noted above and responsible for pancreatic pDC and CXCR3^+^ T cell infiltration in the MRL/MpJ model of AIP are also present in human AIP/IgG4-RD. For this purpose, we determined and compared relevant chemokine concentrations in serum samples collected from healthy controls (*n* = 8) as well as patients with chronic alcoholic pancreatitis (CP) (*n* = 12), and AIP/IgG4-RD patients (*n* = 33) that met the established diagnostic criteria for these disorders (7). We found that serum concentrations of CXCL9, CXCL10, and CCL25 were markedly higher in patients with AIP/IgG4-RD as compared with those with CP and healthy controls, whereas in contrast, serum concentrations of CCL22, a prototypical Th2 chemokine, were not elevated in patients with AIP/IgG4-RD (Figure 11A). We next examined whether serum concentrations of CXCL9, CXCL10, and CCL25 are useful for the assessment of disease activity. Here, we measured serum concentrations of these chemokines before and after successful treatment with prednisolone (PSL). We found successful treatment in patients with AIP/IgG4-RD was associated with a significant reduction in serum concentrations of CXCL9, CXCL10, and CCL25 (Figure 11B). Thus, experimental and human AIP/IgG4-RD are both characterized by enhanced expression of CXCL9, CXCL10, and CCL25 and such enhanced expression is attenuated by treatment.

In previous studies, we reported that serum concentrations of IgG4, IFN-α, and IL-33 were useful biomarkers for diagnosis of AIP/IgG4-RD and monitoring of disease activity (7). We therefore compared levels of these cytokines with levels of relevant chemokines to determine whether the latter might also serve as biomarkers. We found that serum concentrations of IgG4 were positively correlated with those of CXCL9 and CXCL10, but not of CCL25 or CCL22 (Figure 11C). In addition, we found a positive correlation between the serum concentrations of IFN-α and CXCL9 or CXCL10, but no significant correlation between serum concentrations of IFN-α with those of CCL25 or CCL22 (Figure 11D). Finally, we found a positive correlation of serum concentrations of IgG1 and IL-33 with those of CXCL9 or CXCL10 (Supplemental Figures 5 and 6, respectively). Thus, these serum chemokine analyses suggest that serum concentrations of CXCL9, CXCL10, and CCL25 might be useful biomarkers for the diagnosis and monitoring of disease activity in AIP/IgG4-RD.

## Discussion

In previous studies of a murine model of AIP induced in MRL/MpJ mice by repeated administration of poly(I:C) as well as in studies of humans with AIP/IgG4-RD, we provided evidence that AIP/IgG4-RD is caused by pDCs secreting IFN-α (3–5, 7). AIP/IgG4-RD was thus shown to be similar to certain forms of systemic lupus erythematosus (SLE), although in AIP/IgG4-RD, but not in SLE, the pathologic pDC response is marked by secretion of IL-33, a cytokine best known for its support of Th2 responses, but more recently shown to support Th1 responses as well (3, 26). A basic question relating to how AIP/IgG4-RD is initiated and sustained concerns the mechanism of pDC accumulation in the pancreas (or other affected organs) and how such accumulation correlates with the concomitant accumulation of T cells that serve as effector cells in this disease. We now provide answers to this question with studies of the cytokine-induced chemokine ligand/chemokine receptor interactions that underlie the pDC/T cell accumulation occurring in the pancreas during the induction of experimental AIP. Importantly, we show that these interactions form a positive feedback loop that initiates, amplifies, and sustains the pancreatic inflammation; in addition, we show that a key interaction that is part of this loop, that between pDCs and CXCR3^+^ T cells, is necessary for the high-level pDC IFN-α secretion underpinning AIP.

As shown in this study, the initial event causing pancreatitis in the MRL/MpJ model is most likely IFN-α/β production by pancreatic tissue–resident TLR3-bearing cDCs stimulated by a disease-inducing TLR3 ligand such as poly(I:C), rather than IFN-α/β production by stimulated TLR7/9-bearing pDCs. Evidence in support of this conclusion was provided by our demonstration that blockade of TLR3 signaling of cDCs by a TLR3-specific inhibitor abrogates pancreatic inflammation when applied during initial disease induction. Moreover, cDCs isolated from the pancreas at an early time point were the major cellular source of the cytokine/chemokine mixture (type I IFNs, CXCL9, and CXCL10) that enables the next step in the inflammatory process, the pancreatic accumulation of CXCR3^+^ T cells; finally, depletion of CD11c^+^ cDCs, again at an early time point, markedly decreased production of type I IFNs by PMNCs. Based on these observations, we speculate that human AIP/IgG4-RD is initiated by a similar TLR3-bearing cDC-dependent IFN-α/β–inducing mechanism, but in this case the TLR3 stimulus might consist of an organism in the gut microflora (or a product derived from the microflora) that gains access to the pancreas and/or another secretory organ (27). This is suggested by the fact that AIP has been induced in mice by administration of heat-killed, non-pathogenic *Escherichia coli* and that, as discussed more completely below, microbiome abnormalities have been identified in patients with AIP/IgG4-RD (28, 29).

The activation of pancreas-resident TLR3-bearing cDCs by poly(I:C) in the initially uninflamed pancreas is followed by the early appearance of CXCR3-bearing CD4^+^ T cells and the production of Th1 cytokines in the nascent pancreatic inflammation. This is attributable to cDC secretion of IFN-α/β and the latter’s induction of Th1 cells, in this case in lymph nodes draining the pancreas; in addition, it is attributable to cDC production of CXCL9 and CXCL10, chemokines that facilitate chemoattraction of CXCR3-bearing Th1 cells into the pancreas (30). Evidence supporting the occurrence of these cellular events came from the observation that administration of an Ab that blocks CXCL9/CXCL10-CXCR3 interaction prevented migration of IFN-γ–producing Th1 cells into the pancreas and indeed, such Ab administration impeded the development of experimental AIP. Of interest, the recruited CD4^+^CXCR3^+^ T cells do not bear markers of cytolytic cells such as SLAMF7 and granzyme B, as do a proportion of CD4^+^ T cells extracted from human IgG4-RD tissues (9, 17). This may relate to the fact that experimental AIP is a relatively acute inflammation, whereas the human counterpart is a relatively chronic inflammation.

The above IFN-α/chemokine–mediated induction and migration of CXCR3^+^ T cells into the pancreas sets the stage for a second and qualitatively different round of IFN-α/chemokine–mediated cellular influx, i.e., that involving the pancreatic recruitment of pDCs, the main cellular drivers of AIP. Insight into the mechanism of such influx came initially from in vitro studies, in which it was shown that an important additional effect of TLR3 activation of cDCs is inherent in the cDCs’ ability to interact with CXCR3^+^ T cells and thereby to induce T cells that have previously entered the pancreas to produce CCL25, a chemokine with the capacity to cause the chemoattraction and recruitment of CCR9-bearing pDCs into the pancreas. That such CCL25-mediated pDC chemoattraction originating from a cDC–T cell interaction in the early induction phase of experimental AIP (as well as a pDC–T cell interaction in the mature phase of experimental AIP) occurs in vivo and is essential to the progression of the pancreatic inflammation was shown by the fact that administration of anti-CCL25 Ab at the time of poly(I:C) administration prevents production of IFN-α and IL-33 and the development of AIP.

Upon migration into the pancreas and activation at this site, pDCs displace cDCs as the chief cellular drivers of the developing mature AIP. This is evident from previous studies showing that deletion of pDCs during the AIP induction regimen abrogates experimental AIP and that production of IL-33, another proinflammatory cytokine essential to full-blown AIP, is dependent on pDC activation (4, 5). Relating to the necessity for (and advantage of) this switch in the DC type driving AIP, we demonstrated with in vitro studies that pDCs produce large amounts of type I IFNs upon ligand stimulation and CXCR3^+^ T cell interaction; similarly, the amount of CXCR3^+^ T cell–derived CCL25 necessary for further pDC recruitment was much greater in the presence of pDCs than that produced by the same T cell population in the presence of cDCs. Thus, the proinflammatory capacity of pDCs and CXCR3^+^CCL25^+^ T cells during the mature phase of AIP is much greater than that of cDCs and CXCR3^+^CCL25^+^ T cells during the early phase of AIP, and this greater capacity translates into a greater potential to support a more robust pancreatitis. This said, cDCs may continue to contribute to the pancreatic inflammation by their continued production of CXCL9 and CXCL10 since, as also shown in a previous study, the depletion of pDCs does not completely inhibit pancreatic accumulation of CD3^+^ T cells (4).

With the migration of a sufficient number of pDCs into the inflamed pancreas, the cellular elements necessary for the establishment of a positive chemokine feedback loop capable of sustaining AIP are in place. As shown graphically in Figure 10B, this feedback loop consists of pDCs producing CXCL9 and CXCL10 that attract CXCR3^+^ Th1 cells into the pancreas and the latter T cells producing CCL25 that recruits additional CCR9-expressing pDCs with the capacity to renew the cycle and expand the inflammatory milieu. It should be noted, however, that this feedback loop not only serves to stock the pancreas with cellular elements necessary to support AIP, it also serves to enhance the proinflammatory function of the pDCs and T cells subject to recruitment. This became evident from in vitro studies that showed that pDCs required stimulation with TLR9 ligand (CpG) as well as coculture with CXCR3^+^ T cells to produce high levels of IFN-α (and IL-33). In prior studies, IFN-α and/or TNF-α has been shown to upregulate B7 expression and thus to promote the interaction between pDCs and T cells as well as to facilitate pDC activation (31, 32). Thus, the large amounts of IFN-α produced by pDCs and CCL25 produced by CXCR3^+^ T cells in the pancreas of mice displaying AIP could be attributable to enhanced B7/CD28-mediated cellular interaction. However, it is not clear that cell-cell contact is actually necessary for IFN-α production because T cell secretion of CCL25 leads to pDC signaling via CCR9. This idea is favored by the fact that T cells lacking CXCR3 and producing relatively low-level CCL25 secretion did not elicit high-level IFN-α secretion. The possibility that CCL25/CCR9 interaction and signaling has effects on cell function in addition to its effects in chemotaxis is supported by previous studies showing that such interaction promotes tumor cell growth via upregulation of cyclin D1 and tumor cell survival via phosphatidylinositol-3 kinase (PI3K) signaling and Bcl2/Bcl-xl upregulation (33). In addition, it is supported by studies of endothelial cells showing that CCL25-CCR9 interaction causes PI3K activation of Wnt/β-catenin signaling and resulting increased IL-33 secretion (34). This latter finding suggests that CCL25/CCR9 signaling may be responsible for the unique ability of pDCs in AIP/IgG4-RD to produce IL-33.

Another insight into pDC function that became apparent from the in vitro studies conducted here was that pDCs in culture with CXCR3^+^ T cells required TLR9 stimulation (in this case with CpG) to achieve high-level IFN-α and IL-33 secretion. Thus, it is reasonable to ask how such stimulation is acquired in vivo. One possible answer to this question comes from recent studies showing that induction of AIP is suppressed in mice that have undergone gut sterilization; in addition, transfer of fecal microbiota from mice with severe AIP due to high-dose poly(I:C) administration to mice with mild AIP due to low-dose poly(I:C) administration exacerbates pancreatitis in the low-dose–recipient mice (27). Association between murine AIP and dysbiosis has been further explored in studies in which it was shown that intentional induction of compromised intestinal barrier function by oral administration of dextran sodium sulfate led to more severe AIP. Studies of the types of bacteria translocated under these conditions by 16S ribosomal RNA analysis disclosed that whereas many types of bacteria are in fact translocated into the pancreas upon barrier compromise, one organism in particular, *Staphylococcus sciuri*, was associated with more severe pancreatic inflammation and this organism was capable of stimulating pDCs isolated from inflamed tissues to produce increased amounts of IFN-α and IL-33 (35). These studies thus suggest first that induction of pancreatic inflammation results in intestinal dysbiosis and second that CXCR3^+^ T cell–sensitized pDCs driving AIP are being stimulated by TLR9 ligands derived from organisms in the intestinal microflora that gain entry into the pancreas via a compromised intestinal barrier.

Salivary glands are also a frequent site of inflammation in AIP/IgG4-RD (1–3) and, indeed, sialadenitis along with pancreatitis is induced by administration of repeated doses of poly(I:C). This raises the question of whether the cytokine/chemokine positive feedback loop operative in pancreatitis described here is also present in salivary gland with autoimmune sialadenitis. In preliminary studies, we found that *Tlr3* mRNA expression was higher in CD11c^+^ DCs residing in both the uninflamed pancreas and salivary glands than those in the spleen (A Hara, unpublished observations); thus, at least the initial cellular landscape necessary to support such a loop is present in the salivary glands. However, even if IgG4-RD pancreatitis and sialadenitis share pathogenic features it is unlikely that they share the same inductive milieu; in particular, oral dysbiosis is more likely to provide the inducing factor for the development of autoimmune sialadenitis than is gut dysbiosis. This is suggested by the fact that AIP, but not autoimmune sialadenitis, is exacerbated by intestinal barrier disruption (35). In agreement with this idea, autoimmune sialadenitis in Sjogren’s syndrome is linked to oral dysbiosis (36–38). Further studies of the relationship between the oral microbiome and autoimmune sialadenitis will be necessary to more clearly resolve this issue.

Throughout these studies, we have been considering CD4^+^CXCR3^+^ T cells as Th1 cells, but it is important to point out that the CD4^+^CXCR3^+^ T cell subset includes Tfh1 cells as well and that there is evidence that type I IFNs also induce Tfh1 cells (11, 13, 16, 39–41). Given the role of Tfh cells in B cell responses, possible induction of Tfh cells is important because it provides a cellular mechanism to explain how the excessive pDC function interrelates with the genesis of a major feature of AIP/IgG4-RD, namely germinal center formation and the production of autoAbs (10). With respect to the latter, there is emerging evidence that while IgG4 elevations are diagnostic markers of AIP/IgG4-RD (1–3), IgG1 rather than IgG4 autoAbs are the actual Abs causing pathology in humans with this disease (42–44). In addition, it seems likely that pDC production of IFN-α and the latter’s induction of Th1/Tfh1 T cells can be a contributor to immunopathogenesis of human AIP/IgG4-RD because in humans, IgG4 and IgG1 Ab responses are under Th2/Tfh2 and Th1/Tfh1 regulation, respectively (45).

In previous studies, we found that serum IFN-α and IL-33 concentrations can be useful biomarkers not only for diagnosis, but also for disease activity monitoring in AIP/IgG4-RD (7). In particular, we saw a strong positive correlation between serum concentrations of IgG4 and those of IFN-α or IL-33. In the present study, we found that CXCL9, CXCL10, and CCL25 could also serve as biomarkers for AIP/IgG4-RD inasmuch as serum concentrations of these factors were markedly higher in patients AIP/IgG4-RD than in those with CP or healthy controls. In yet another biomarker comparison, we found that serum levels of IgG4 and IFN-α were positively correlated with those of CXCL9 and CXCL10, but not with CCL25, in individual patients with AIP/IgG4-RD. The latter lack of correlation could be due to the possibility that AIP/IgG4-RD observed in humans is a more chronic inflammation than is experimental murine AIP; thus, in AIP/IgG4-RD there might be a plateau effect wherein pDC activity is limited not by degree of cell accumulation, but rather by the level of TLR ligand stimulation. Taken as a whole, the fact that elevations in the chemokine elements observed in the MRL/MpJ model of AIP are also seen in humans with AIP/IgG4-RD provides support for the idea that the chemokine/cytokine cascade driving disease in the murine model is also functional in the human disease. However, given the greater chronicity and multiorgan complexity of human AIP/IgG4-RD, studies of chemokine levels in larger groups of patients over longer periods of time are necessary to establish this possibility. Such studies not only have the potential to provide new biomarkers of AIP/IgG4-RD, but also to identify possible targets for treatment of AIP/IgG4-RD that have minimal effects on overall immune competence and resistance to infection.

## Methods

### Sex as a biological variable.

Our study exclusively examined female mice. Further investigation is required to evaluate whether the findings are relevant for male mice.

### Reagents and Abs.

Details of reagents and Abs, including the sources, catalog number, and clone names are shown in Supplemental Table 1.

### Induction of AIP in MRL/MpJ mice.

Female 6-week-old MRL/MpJ mice (Japan SLC) were maintained in the Kindai University animal facility under specific pathogen–free conditions. MRL/MpJ mice were treated with intraperitoneal injection of 100 μg of poly(I:C) (InvivoGen) twice a week for a total of 16 times to induce experimental AIP (4, 5, 27, 35).

### Blockade of TLR3 signaling pathways.

Blockade of TLR3-mediated signaling pathways was performed as previously described (18). The TLR3/dsRNA complex inhibitor [(*R*)-2-(3-chloro-6-fluorobenzo(b)thiophene-2-carboxamido)-3-phenylpropanoic acid, Calbiochem] was dissolved in DMSO and diluted in saline. The inhibitor was administered by intraperitoneal injection; 1 mg of the inhibitor was injected prior to each poly(I:C) administration.

### Neutralization of signaling pathways mediated by IFN-α, IL-33, CXCR3, and CCL25.

Neutralization of IFN-α and IL-33 or the depletion of pDCs was performed as previously described (4, 5). Briefly, in some studies, MRL/MpJ mice were administered anti-IFNAR Ab (100 μg, BD Biosciences), anti–IL-33 receptor ST2 Ab (anti-ST2 Ab, 100 μg, R&D Systems), or anti–bone marrow stromal cell Ag 2 Ab (120G8 Ab, 100 μg, Dendritics) prior to administration of each poly(I:C) injection, whereas in other studies mice were administered anti-CXCR3 Ab (200 μg, Bio X Cell), anti-CCL2 Ab (100 μg, Bio X Cell), or anti-CCL25 Ab (100 μg, R&D Systems) prior to each poly(I:C) injection. Control mice were administered 100 μg mouse IgG (Sigma-Aldrich), 200 μg hamster IgG (Bio X Cell), 100 μg rat IgG (Sigma-Aldrich), or 100 μg goat IgG (Invitrogen).

### Flow cytometric analysis and sorting.

PMNCs were isolated from the pancreas of MRL/MpJ mice treated with poly(I:C) as previously described (4, 5, 27). PMNCs were stained with FITC-, Alexa Fluor 488–, or PE-conjugated anti-B220 Ab (Invitrogen), anti–PDCA-1 Ab (Invitrogen), anti-CD3 Ab (Invitrogen), anti-CD4 Ab (BioLegend), anti-CD8 Ab (BioLegend), anti-CD11b Ab (Invitrogen), anti-CD11c Ab (Invitrogen), anti-CCR4 Ab (BioLegend), and anti-CXCR3 Ab (R&D Systems). In some experiments, PMNCs were stained with Alexa Fluor 647–conjugated anti-CCR2 Ab, anti-CCR7 Ab, and anti-CCR9 Ab (all from BioLegend) in combination with PE-conjugated anti–PDCA-1 Ab and FITC-conjugated anti-B220 Ab. Alexa Fluor 647–conjugated rat IgG2a or IgG2b (both from BioLegend) was used as a isotype-matched control Ab. To examine cell-surface expression of SLAMF7, PMNCs were stained with APC-conjugated anti-SLAMF7 Ab (BioLegend) in combination with Alexa Fluor 488–conjugated anti-CD4 Ab (BioLegend) and PE-conjugated anti-CXCR3 Ab (R&D Systems). Intracellular staining of granzyme B was performed using APC-conjugated anti–granzyme B Ab (BioLegend) in combination with Alexa Fluor 488–conjugated anti-CD4 Ab (BioLegend) and PE-conjugated anti-CXCR3 Ab (R&D Systems). APC-conjugated rat IgG1 Ab (BioLegend) was used as an isotype control Ab for SLAMF7 and granzyme B staining. Flow cytometric analysis was performed by using an Accuri C6 flow cytometer (BD Biosciences) and CFlow Plus software (BD Biosciences).

pDCs (defined as PDCA-1^+^B220^lo^ cells), CD3^+^CXCR3^+^ T cells, and CD3^+^CXCR3^-^ T cells were FACS isolated by using APC-conjugated anti–PDCA-1 Ab (BioLegend), FITC-conjugated anti-B220 Ab (Invitrogen), PerCP-Cy5.5–conjugated anti-CD3 Ab (Biolegend), and PE-conjugated anti-CXCR3 Ab (R&D Systems), as previsouly described (35). In some experiments, CD11c^+^ DCs, CD3^+^CXCR3^+^ T cells, and CD3^+^CXCR3^–^ T cells were FACS isolated by using APC-conjugated anti-CD11c Ab (BioLegend), FITC-conjugated anti-CD3 Ab (BioLegend), and PE-conjugated anti-CXCR3 Ab (R&D Systems). FACS was performed by using a FACSAria (BD Biosciences). The purity of these cells was greater than 99%. CD3^+^CXCR3^+^ T cells (1 × 10^5^/mL) or CD3^+^CXCR3^-^ T cells (1 × 10^5^/mL) were cocultured with pDCs (1 × 10^5^/mL) or CD11c^+^ DCs (1 × 10^5^/mL). T cells were stimulated with anti-CD3 Ab (5 μg/mL, 2C11, BD Biosciences). pDCs were stimulated with CpG1585 (1 μM, InvivoGen). In some experiments, anti-IFNAR Ab (100 μg/mL, R&D Systems) or goat IgG (100 μg/mL, Invitrogen) was used. Cell culture supernatants obtained at 48 hours were analyzed by enzyme-linked immunosorbent assay (ELISA) to determine the concentrations of IFN-α and CCL25.

### qPCR analysis and culture.

cDCs and pDCs were isolated from the pancreas and spleen of MRL/MpJ mice. pDCs and cDCs were FACS isolated by using APC-conjugated anti–PDCA-1 Ab (BioLegend), PerCP-Cy5.5–conjugated anti-B220 Ab (BioLegend), BV421-conjugated anti-IA/IE Ab (BioLegend), and BV711-conjugated anti-CD11c Ab (BioLegend). CD11c^+^IA/IE^+^ cells were defined as cDCs. mRNA was isolated from FACS-isolated pDCs and cDCs by using an RNeasy Mini kit (Qiagen). Expression of *Tlr3* and *Tlr9* mRNA was determined by qPCR analyses using primers purchased from Qiagen, as previously described (46). In some experiments, sorted pancreatic pDCs (1 × 10^6^/mL) and cDCs (1 × 10^6^/mL) were stimulated with poly(I:C) (25 μg/mL) or CpG (1 μM) for 48 hours to measure production of IFN-α, IFN-β, CXCL9, and CXCL10. The CD11c^+^ DC–depleted cell fraction was prepared from whole PMNCs using anti-CD11c microbeads (Miltenyi Biotec). The purity was greater than 90%. Whole PMNCs (1 × 10^6^/mL) and CD11c^+^ cell–depleted PMNCs (1 × 10^6^/mL) were stimulated with poly(I:C) (25 μg/mL) for 48 hours to measure production of IFN-α and IFN-β.

### Cytokine and chemokine assays.

Pancreatic concentrations of cytokines and chemokines were measured as previously described (47). Briefly, tissues were cut into small pieces by scissors and then homogenized with a homogenizer. Protein extracts were prepared using a nuclear extraction kit from Active Motif. After protein concentrations were determined, isolated protein extracts were analyzed by ELISA. Pancreatic concentrations of murine IFN-α, IFN-β, CXCL9, CXCL10, CCL17, and CCL22 were measured using ELISA kits from R&D Systems. Pancreatic concentrations of murine CCL25, IL-33, IL-4, IL-17, IFN-γ, and TNF-α were measured using ELISA kits from Invitrogen, as previously described (47). Serum concentrations of human IFN-α, IL-33, CXCL9, CXCL10, and CCL22 were measured using ELISA kits from R&D Systems, as previously described (7). Serum concentrations of CCL25 was measured using an ELISA kit from Invitrogen.

### Immunohistochemical and immunofluorescence analyses.

Formalin-fixed pancreatic tissues were deparaffinized and subjected to immunohistochemical and immunofluorescence analyses, as previously described (4, 5, 47). Hematoxylin and eosin–stained pancreatic samples were used to perform pathological assessment, as previously described (4, 5). Pancreatic inflammation was scored as follows: 0, pancreas without mononuclear cell infiltration; 1, mononuclear cell aggregation and/or infiltration within the interstitium with no parenchymal destruction; 2, focal parenchymal destruction with mononuclear cell infiltration; 3, diffuse parenchymal destruction, but some intact parenchymal areas retained; 4, almost all pancreatic tissue, except the pancreatic islets, destroyed or replaced with fibrosis or adipose tissue. Two representative images were acquired for each sample and the average value of pathology scores was regarded as the value of each sample. Immunofluorescent staining was performed using rat anti-CD3 Ab (Abcam) and rabbit anti-CCL25 Ab (Novus Biologicals) followed by incubation with Alexa Fluor 488–conjugated anti-rat IgG or Alexa Fluor 546–conjugated anti-rabbit IgG (Invitrogen). CCL25 expression was visualized by using anti–CCL25 Ab (Novus Biologicals) as primary Ab and Dako EnVision+ system (Dako Japan), as previously described (47). Numbers of CCL25^+^ lymphoid cells in high-powered fields were manually counted in 2 representative images from each sample. Cell counting was performed in a blinded manner and the average value from 2 representative images was regarded as the value of each sample.

### Human samples.

Serum samples were obtained from patients with CP (*n* = 12) or AIP/IgG4-RD (*n* = 33), as previously described (7). Eight samples obtained from healthy controls were used as reference serum. Thirty-three patients who fulfilled the diagnostic criteria for definite type 1 AIP and/or IgG4-RD were included (48, 49). Twelve patients who fulfilled the definite criteria for the diagnosis of CP were also included (50). Serum samples were obtained at the diagnosis of AIP/IgG4-RD or CP.

### PSL treatment.

Fourteen patients with AIP/IgG4-RD were treated with oral administration of PSL for the induction of remission, as previously described (7). Successful induction of remission was confirmed by positron emission tomography/computed tomography.

### Statistics.

An unpaired, 2-tailed Student’s *t* test was used to evaluate the differences between 2 groups of parameters. The Kruskal-Wallis test (a nonparametric version of 1-way ANOVA) was used to evaluate the differences between more than 2 groups. For post hoc analysis, the Bonferroni-corrected Mann-Whitney *U* test was performed for comparison between 2 groups, as previously described (7). Wilcoxon’s signed- rank test was used to compare serum chemokines before and after PSL treatment. Spearman’s correlation coefficient was used to determine the association between the different serum markers evaluated in this study. All statistical analyses were performed using Prism 10 (GraphPad Software Inc.). *P* values of less than 0.05 indicated statistical significance.

### Study approval.

The protocols of animal experiments were approved by the Review Boards of the Kindai University Institutional Animal Care and Use Committee (Osaka-Sayama, Osaka, Japan). All human participants were enrolled in accordance with Declaration of Helsinki principles and using protocols approved by the institutional review board of Kindai University Faculty of Medicine (Osaka-Sayama, Osaka, Japan, approval number: 28-034). The study protocol conformed to the ethical guidelines for human clinical research established by the Japanese Ministry of Health, Labor and Welfare and written informed consent was obtained from all patients and healthy control individuals at enrollment.

### Data availability.

Raw data are available in the supplemental Supporting Data Values XLS file.

## Author contributions

AH, TW, and WS conceptualized the study. AH, TW, KM, TY, M Kurimoto, IS, YM, RT, YO, KK, and ST developed the methodology. AH, TW, KM, TY, M Kurimoto, IS, YM, RT, YO, KK, and ST performed experiments. TW, KM, and KK acquired funding. M Kudo and WS supervised the study. AH, TW, KM, and WS wrote the original draft of the manuscript, which was reviewed and edited by AH, TW, and WS.

## Supplementary Material

Supplemental data

Supporting data values

## Version 1 09/26/2024

In-Press Preview

## Version 2 10/22/2024

Electronic publication
