# Supplementary material for: A positive cytokine/chemokine feedback loop establishes plasmacytoid DC–driven autoimmune pancreatitis in IgG4-related disease
Source: JCI Insight. 2024 Sep 12;9(20):e167910. doi: 10.1172/jci.insight.167910 (PMC11529986; doi:10.1172/jci.insight.167910)
Supplement: Supplemental data [file jciinsight-9-167910-s060.pdf]

**Supplemental Material**

# A Positive Cytokine/Chemokine Feedback Loop Establishes Plasmacytoid Dendritic Cell-Driven Autoimmune Pancreatitis in IgG4-Related Disease

Akane Hara[1*], Tomohiro Watanabe[1*], Kosuke Minaga[1], Tomoe Yoshikawa[1], Masayuki Kurimoto[1], Ikue Sekai[1], Yasuhiro Masuta[1], Ryutaro Takada[1], Yasuo Otsuka[1], Ken Kamata[1], Shiki Takamura[2], Masatoshi Kudo[1], Warren Strober[3]
*These authors contributed equally to this work.

## Author affiliations

[1]Department of Gastroenterology and Hepatology, Kindai University Faculty of Medicine, Osaka-Sayama, Osaka, Japan
[2]Laboratory for Immunological Memory, RIKEN IMS Center for Integrative Medical Science, Yokohama, Japan
[3]Mucosal Immunity Section, Laboratory of Host Defenses, National Institute of Allergy and Infectious Diseases, National Institutes of Health, Bethesda, MD, USA.

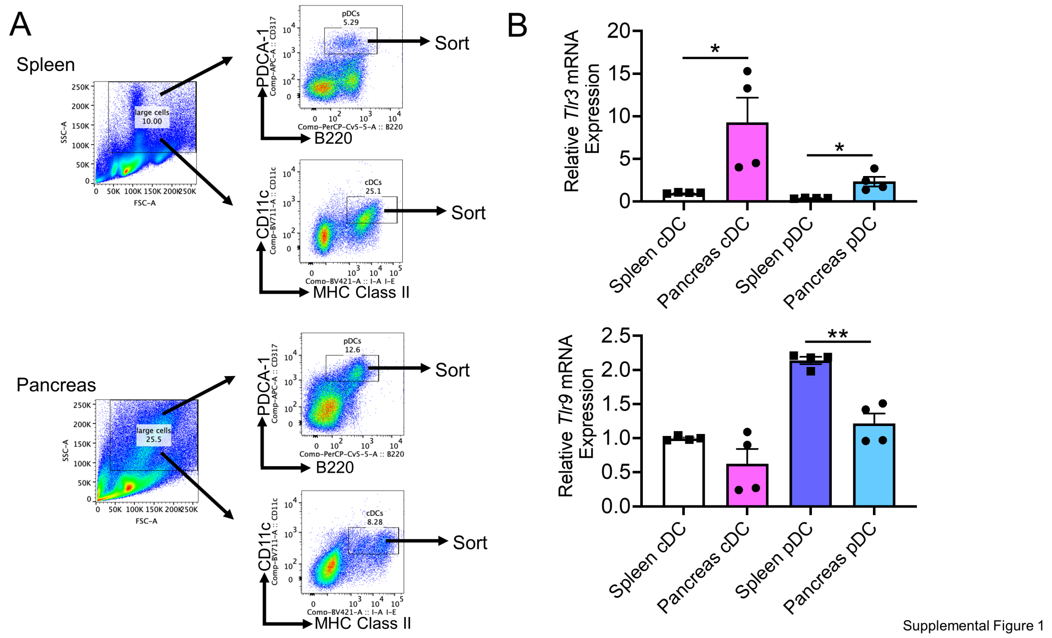


**Supplemental Figure 1. mRNA expression of *Tlr3* and *Tlr9* in conventional and plasmacytoid dendritic cells.**

**(A)** FACS-sorting gating strategies used for isolation of plasmacytoid dendritic cells (pDCs) and conventional DCs from pancreases and spleens of untreated MRL/MpJ mice (n=4). **(B)** Expression of *Tlr3* and *Tlr9* mRNA in each DC subset determined by quantitative reverse transcription polymerase chain reaction. Each dot corresponds to the value in an individual mouse. Results shown are combined data of two independent experiments and are expressed as means + standard error. Statistical analyses were performed using unpaired two-tailed Student's t test. *P<0.05, **P<0.01.

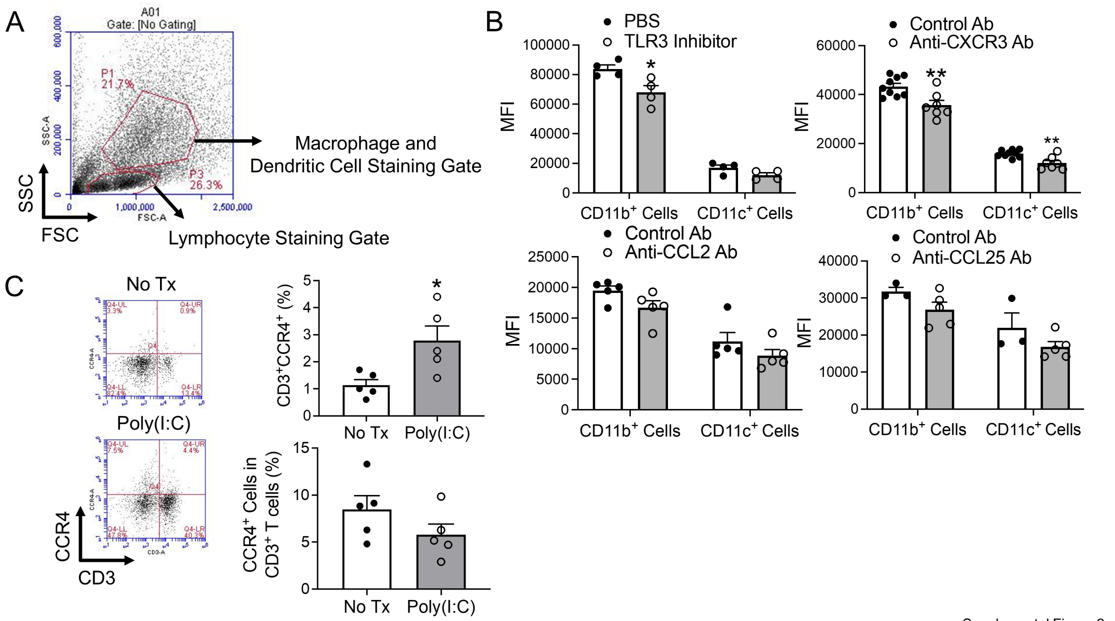


Supplemental Figure 2

**Supplemental Figure 2. Percentages of CD3$^+$C-C chemokine receptor 4$^+$ T cells in the pancreas.**

Groups of MRL/MpJ mice were treated as described in Figures 1, 3, 4, 6, and 7. After sacrifice pancreases were processed for acquisition of pancreatic mononuclear cells (PMNCs). **(A)** Flowcytometry gating for analysis of stained lymphocytes, or macrophages and dendritic cells among PMNCs. **(B)** Mean fluorescence intensity (MFI) in CD11b$^+$ or CD11c$^+$ cells. **(C)** Groups of MRL/MpJ mice were treated as described in Figure 3. After sacrifice pancreases were processed for acquisition of PMNCs. The percentages of pancreatic CD3$^+$C-C chemokine receptor 4 (CCR4)$^+$ T cells within PMNCs and the percentages of CCR4$^+$ cells within CD3$^+$ T cells; left panel: representative flowcytometric analyses; right panel: bar graphs of cumulative data from individual mice. Each dot in (B) and (C) corresponds to the value in individual mice. Statistical analyses were performed using unpaired two-tailed Student's t test. Results are expressed as means + standard error. *P<0.05, **P<0.01.

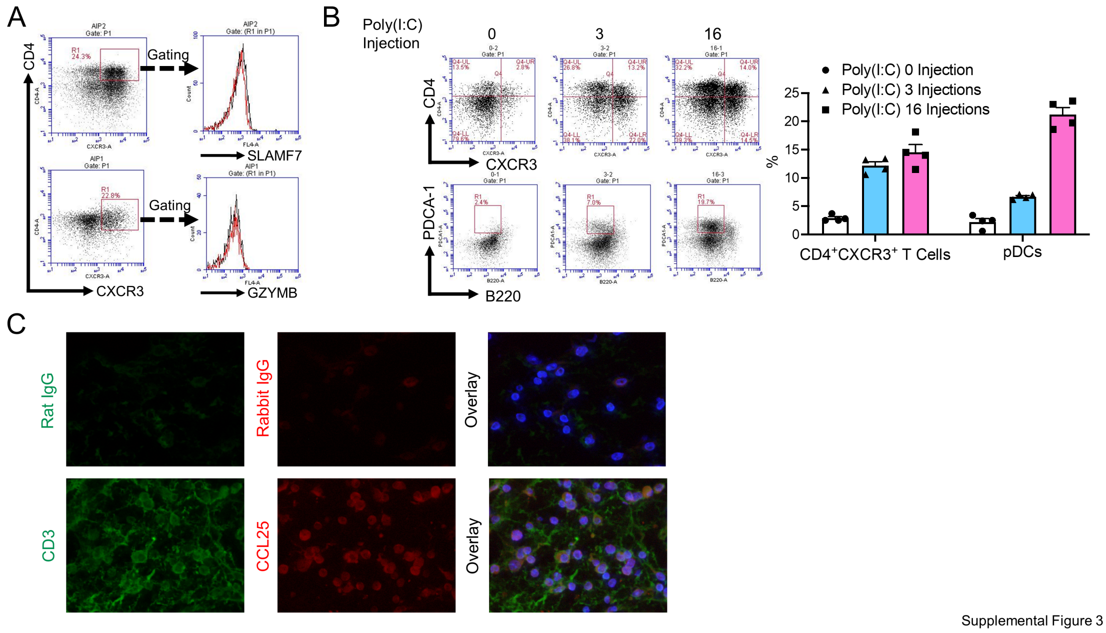


Supplemental Figure 3

**Supplemental Figure 3. Early and late accumulation of CD4$^{+}$C-X-C motif chemokine receptor 3$^{+}$ T cells and plasmacytoid dendritic cells in the pancreas during autoimmune pancreatitis development.**

**(A)** MRL/MpJ mice were treated as described in Figure 3. After sacrifice pancreases were processed for acquisition of pancreatic mononuclear cells (PMNCs). PMNCs were stained with Alexa Fluor 488-conjugated anti-CD4 Ab, PE-conjugated anti-C-X-C motif chemokine receptor 3 (CXCR3) Ab, in combination with APC-conjugated anti-SLAMF7 Ab or APC-conjugated anti-granzyme B (GZYMB) Ab. Cells were then subjected to gated flowcytometric analysis as indicated to determine expression of cell-surface SLAMF7 and intracellular GZYMB (right panels; control Ab, black lines, anti-SLAMF7 Ab or anti-GZYMB Ab, red lines). **(B)** Groups of MRL/MpJ mice were treated as described in Figure 3, but in this case PMNCs were extracted from polyinosinic-polycytidylic acid (poly(I:C))-treated mice for a total of three times (n=4) or 16 times (n=4); in addition, PMNCs were extracted from untreated MRL/MpJ mice (n=4) to obtain base line data. PMNCs were stained with PE-conjugated anti-PDCA-1 Ab and FITC-conjugated anti-B220 Ab and were stained with PE-conjugated anti-CXCR3 Ab and Alexa Fluor 488-conjugated anti-CD4 Ab to evaluate accumulation of pDCs and CD4$^{+}$CXCR3$^{+}$ T cells, respectively. Left panel: representative flowcytometric analyses; right panel: bar graphs of cumulative data from individual mice. Each dot corresponds to the value in one mouse. Results are expressed as means + standard error. **(C)** MRL/MpJ mice

were treated as described in Figure 3. After sacrifice mouse pancreases were processed for tissue fixation and staining with anti-C-C motif chemokine ligand 25 (CCL25) Ab (red fluorescence) and anti-CD3 Ab (green fluorescence); rat IgG and rabbit IgG were used as isotype-matched control Abs. Results shown are the representative images in three independent experiments. Magnification X1200.

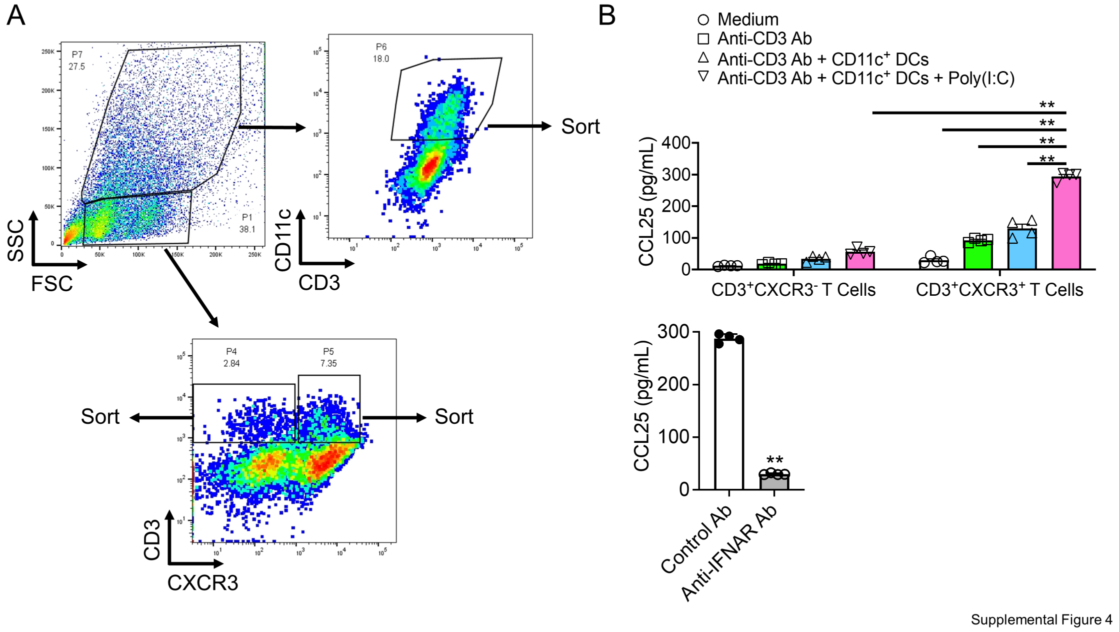


**Supplemental Figure 4. CD3$^{+}$C-X-C motif chemokine receptor 3$^{+}$ T cells produce C-C motif chemokine ligand 25 in response to IFN-$\alpha$ secreted by CD11c$^{+}$ dendritic cells.**

**(A)** FACS-sorting gating strategy for acquisition of CD3$^{+}$C-X-C motif chemokine receptor 3 (CXCR3)$^{+}$ T cells, CD3$^{+}$CXCR3$^{-}$ T cells, and CD11c$^{+}$ dendritic cells (DCs). **(B)** Pancreatic mononuclear cells (PMNCs) were isolated from pancreases of MRL/MpJ mice (n=3) administered polyinosinic-polycytidylic acid (poly(I:C)) twice a week for a total of 3 times by intraperitoneal injection. PMNCs were subjected to FACS-sorting to acquire purified CD3$^{+}$CXCR3$^{+}$ T cells, CD3$^{+}$CXCR3$^{-}$ T cells, and CD11c$^{+}$ DCs after which they were cultured for 48 hours (at 1X10$^{5}$/mL) in quadruplicate as indicated in the presence of anti-CD3 Ab (5 $\mu$g/mL) alone or with poly(I:C)(25 $\mu$g/mL); in addition, in some experiments, anti-IFN $\alpha\beta$ receptor Ab (anti-IFNAR Ab, 100 $\mu$g/mL) or control Ab was added to the culture. Cell culture supernatants obtained from each well were subjected to enzyme-linked immunosorbent assay for measurement of CCL25. Each dot represents the CCL25 concentration value derived from each well. Results shown are representative one of two independent experiments and are expressed as means + standard error. Statistical analyses were performed using Kruskal–Wallis test and Bonferroni-corrected Mann-Whitney U test. **P<0.01.

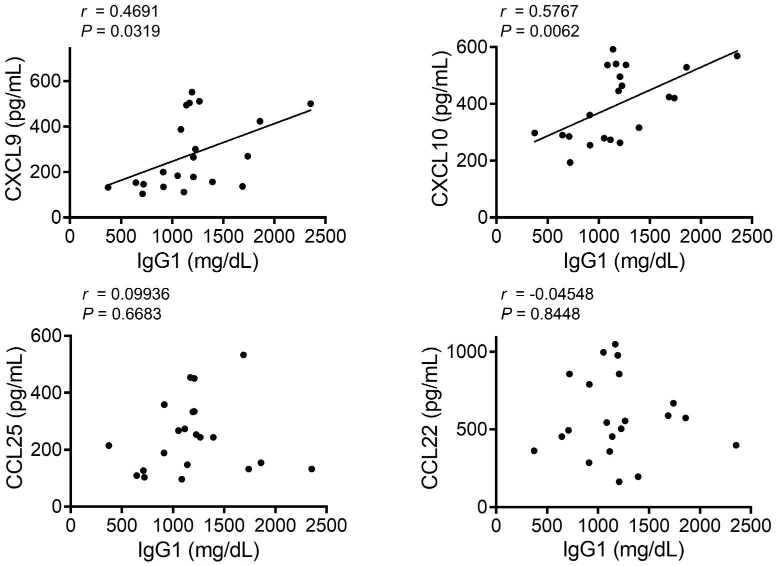


Supplemental Figure 5

**Supplemental Figure 5. Co-relation between serum IgG1 levels and those of chemokines in patients with autoimmune pancreatitis and IgG4-related disease.**

Serum samples of patients with autoimmune pancreatitis and IgG4-related disease (AIP/IgG4-RD) were prepared as described in Figure 11. Serum levels of IgG1, C-X-C motif chemokine ligand 9 (CXCL9), CXCL10, C-C motif chemokine ligand 25 (CCL25), and CCL22 in patients with AIP/IgG4-RD were measured by enzyme-linked immunosorbent assay. Each dot represents one patient. P-values and correlation coefficient (r) values, as determined by the Spearman’s rank correlation test.

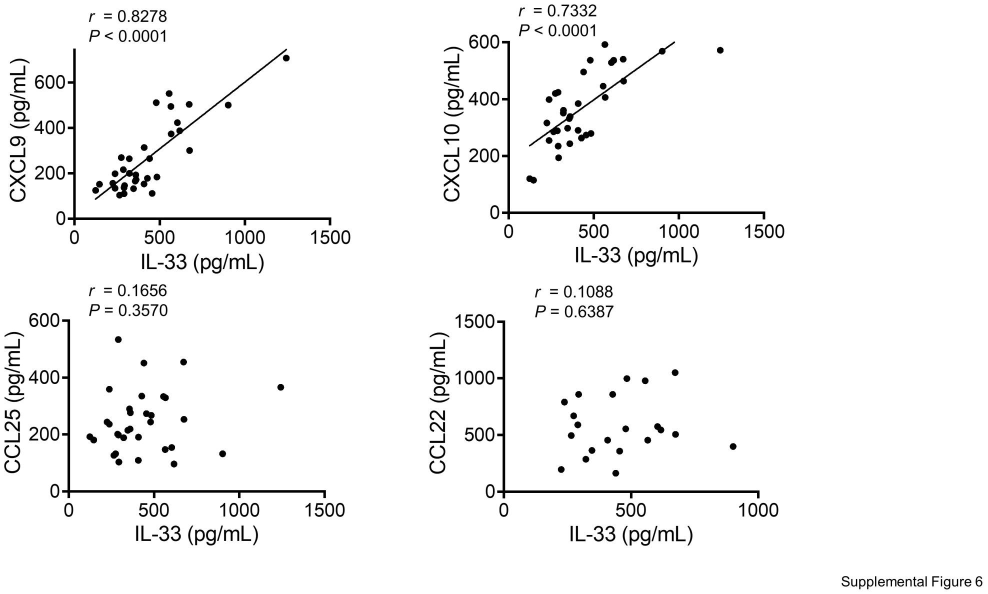


**Supplemental Figure 6. Co-relation between serum IL-33 levels and those of chemokines in patients with autoimmune pancreatitis and IgG4-related disease.**

Serum samples of patients with autoimmune pancreatitis and IgG4-related disease (AIP/IgG4-RD) were prepared as described in Figure 11. Serum levels of IL-33, C-X-C motif chemokine ligand 9 (CXCL9), CXCL10, C-C motif chemokine ligand 25 (CCL25), and CCL22 in patients with AIP/IgG4-RD were measured by enzyme-linked immunosorbent assay. Each dot represents one patient. P-values and correlation coefficient (r) values, as determined by Spearman's rank correlation test, are shown.

## Supplemental Table 1. Reagents, sources and identifier.

| TLR ligands and inhibitors | Sources | Identifier |
|---|---|---|
| TLR3/ds RNA complex inhibitor | Calbiochem | Cat#614310 |
| Poly(I:C) | InvivoGen | Cat#tlrl-pic |
| CpG | InvivoGen | Cat#tlrl-1585 |
| **PCR primers** | | |
| **Target** | **Sources** | **Identifier** |
| mouse TLR3 | Qiagen | Cat#QT00122983 |
| mouse TLR9 | Qiagen | Cat#QT01043049 |
| **Abs for *in vivo* and *in vitro* studies** | | |
| **Ab (Clone)** | **Sources** | **Identifier** |
| Anti-mouse BST2 Ab (120G8) | Dendritics | Cat#DDX0390 |
| Anti-mouse IFNAR Ab (MAR1-5A3) | BD Biosciences | Cat#561183 |
| Anti-mouse ST2 Ab (245707) | R&D Systems | Cat#MAB10041 |
| Anti-mouse CXCR3 Ab (CXCR3-173) | Bio X Cell | Cat#BE0249 |
| Anti-mouse CCL2 Ab (2H5) | Bio X Cell | Cat#BE0185 |
| Anti-mouse CCL25 Ab (Polyclonal) | R&D Systems | Cat#AF-481-NA |
| Hamster IgG (Isotype Control Ab) | Bio X Cell | Cat#BE0091 |
| Rat IgG (Isotype Control Ab) | Sigma-Aldrich | Cat#14131 |
| Mouse IgG (Isotype Control Ab) | Sigma-Aldrich | Cat#15381 |
| Goat IgG (Isotype Control Ab) | Invitrogen | Cat#02-6202 |
| Anti-mouse IFN-$\alpha/\beta$ receptor Ab (Polyclonal) | R&D Systems | Cat#AF3039 |
| Goat IgG (Isotype Control Ab) | Invitrogen | Cat#02-6202 |
| Anti-mouse CD3 Ab (145-2C11) | Invitrogen | Cat#16-0031-82 |
| **Abs for immunofluorescence and immunohistochemical studies** | | |
| **Ab (Clone)** | **Sources** | **Identifier** |
| Anti-mouse CCL25 Ab (Polyclonal) | Novus Biologicals | Cat#NBP1-40993 |
| Anti-mouse CD3 Ab (CD3-12) | Abcam | Cat#ab11089 |
| Alexa Fluor 546-Anti-Rabbit IgG Ab | Invitrogen | Cat#A11010 |
| Alexa Fluor 488-Anti-Rat IgG Ab | Invitrogen | Cat#A21210 |
| Rat IgG (Isotype Control Ab) | Sigma-Aldrich | Cat#14131 |
| Rabbit IgG (Isotype Control Ab) | Sigma-Aldrich | Cat#12-370 |
| **MACS beads** | | |
| Anti-mouse CD11c Microbeads Ultrapure | Miltenyi Biotec | Cat#130-125-835 |

**Supplemental Table 1. Continued.**

| ELISA kits | | |
|---|---|---|
| **Mouse** | **Sources** | **Identifier** |
| CCL2 | Invitrogen | Cat#88-7391-22 |
| CCL17 | R&D Systems | Cat#MCC170 |
| CCL22 | R&D Systems | Cat#MCC220 |
| CCL25 | Invitrogen | Cat#EMCCL25 |
| CXCL9 | R&D Systems | Cat#MCX900 |
| CXCL10 | R&D Systems | Cat#DY466 |
| IFN-$\alpha$ | R&D Systems | Cat#42120-1 |
| IFN-$\beta$ | R&D Systems | Cat#42400-1 |
| IFN-$\gamma$ | Invitrogen | Cat#88-7314-88 |
| IL-4 | Invitrogen | Cat#88-7044-22 |
| IL-17 | Invitrogen | Cat#88-7371-22 |
| IL-33 | Invitrogen | Cat#88-7333-88 |
| TNF-$\alpha$ | Invitrogen | Cat#88-7324-88 |
| **Human** | **Sources** | **Identifier** |
| CCL22 | R&D Systems | Cat#DMD00 |
| CCL25 | Invitrogen | Cat#EHCCL25 |
| CXCL9 | R&D Systems | Cat#DCX900 |
| CXCL10 | R&D Systems | Cat#DIP100 |
| IFN-$\alpha$ | R&D Systems | Cat#41100-1 |
| IL-33 | R&D Systems | Cat#D3300B |

**Supplemental Table 1. Continued.**

| **Ab for flowcytometric analysis and FACS sorting** | | | |
|---|---|---|---|
| **Ab (Clone)** | **Fluorochrome** | **Sources** | **Identifier** |
| Anti-mouse B220 Ab (RA3-6B2) | FITC | Invitrogen | Cat#11-0452-82 |
| Anti-mouse B220 Ab (RA3-6B2) | PerCP-Cy5.5 | BioLegend | Cat#103235 |
| Anti-mouse CCR2 Ab (SA203G11) | Alexa Fluor 647 | BioLegend | Cat#150603 |
| Anti-mouse CCR4 Ab (2G12) | PE | BioLegend | Cat#131203 |
| Anti-mouse CCR7 Ab (4B12) | Alexa Fluor 647 | BioLegend | Cat#120111 |
| Anti-mouse CCR9 Ab (9B1) | Alexa Fluor 647 | BioLegend | Cat#129709 |
| Anti-mouse CD3 Ab (17A2) | FITC | BioLegend | Cat#100204 |
| Anti-mouse CD3 Ab (145-2C11) | PE | Invitrogen | Cat#12-0031-82 |
| Anti-mouse CD3 Ab (145-2C11) | PerCP-Cy5.5 | BioLegend | Cat#100327 |
| Anti-mouse CD4 Ab (GK1.5) | Alexa Fluor 488 | BioLegend | Cat#100425 |
| Anti-mouse CD8 Ab (53-6.7) | Alexa Fluor 488 | BioLegend | Cat#100726 |
| Anti-mouse CD11b Ab (M1/70) | FITC | Invitrogen | Cat#11-0112-82 |
| Anti-mouse CD11c Ab (N418) | PE | Invitrogen | Cat#12-0114-81 |
| Anti-mouse CD11c Ab (N418) | BV711 | BioLegend | Cat#117349 |
| Anti-mouse CD11c Ab (N418) | APC | BioLegend | Cat#117309 |
| Anti-mouse CXCR3 Ab (220803) | PE | R&D Systems | Cat#FAB1685P |
| Anti-human/mouse Granzyme B Ab (QA18A28) | APC | BioLegend | Cat#396407 |
| Anti-mouse I-A/I-E Ab (M5/114.15.2) | BV421 | BioLegend | Cat#107632 |
| Anti-mouse PDCA-1 Ab (eBio927) | PE | Invitrogen | Cat#12-3172-81 |
| Anti-mouse PDCA-1 Ab (927) | APC | BioLegend | Cat#127015 |
| Anti-mouse SLAMF7 Ab (4G2) | APC | BioLegend | Cat#152003 |
| Rat IgG1 (Isotype Control Ab) | APC | BioLegend | Cat#400411 |
| Rat IgG2a (Isotype Control Ab) | Alexa Fluor 647 | BioLegend | Cat#400526 |
| Rat IgG2b (Isotype Control Ab) | Alexa Fluor 647 | BioLegend | Cat#400626 |
